# Risk of Suicide and Psychiatric Disorders Among Isotretinoin Users: A Meta-Analysis

**DOI:** 10.1001/jamadermatol.2023.4579

**Published:** 2023-11-29

**Authors:** Nicole Kye Wen Tan, Adelina Tang, Neil Chen Yi Lun MacAlevey, Benjamin Kye Jyn Tan, Hazel H. Oon

**Affiliations:** 1 Yong Loo Lin School of Medicine, National University of Singapore, Singapore; 2 National Skin Centre and Skin Research Institute, Singapore

## Abstract

**Importance:**

Isotretinoin is hypothesized to contribute to the development of psychiatric disorders, but the epidemiological association and risk factors associated with psychiatric disorders among isotretinoin users remain unclear.

**Objective:**

To clarify the absolute and relative risk and risk factors associated with suicide and psychiatric disorders among isotretinoin users.

**Data Sources:**

PubMed, Embase, Web of Science, and Scopus were searched from inception until January 24, 2023.

**Study Selection:**

Randomized trials and observational studies were selected if they reported the absolute risk, relative risk, and risk factors for suicide and psychiatric disorders among isotretinoin users.

**Data Extraction and Synthesis:**

Relevant data were extracted and risk of bias was evaluated at the study level using the Newcastle-Ottawa Scale. Data were pooled using inverse variance-weighted meta-analyses. Heterogeneity was measured using the *I*^2^ statistic, and meta-regression analyses were performed.

**Main Outcomes and Measures:**

Absolute risk (percentage), relative risks (risk ratios [RR]), and risk factors (RR) of suicide and psychiatric disorders among isotretinoin users.

**Results:**

A total of 25 studies including 1 625 891 participants were included in the review and 24 in the meta-analysis. Among the included studies, participants’ average age ranged from 16 to 38 years, and distribution by sex ranged from 0% to 100% male. The 1-year pooled absolute risk from between 2 and 8 studies of completed suicide, suicide attempt, suicide ideation, and self-harm were each less than 0.5%, while that of depression was 3.83% (95% CI, 2.45-5.93; *I*^2^ = 77%) in 11 studies. Isotretinoin users were less likely than nonusers to attempt suicide at 2 years (RR, 0.92; 95% CI, 0.84-1.00; *I*^2^ = 0%), 3 years (RR, 0.86; 95% CI, 0.77-0.95; *I*^2^ = 0%), and 4 years (RR, 0.85; 95% CI, 0.72-1.00; *I*^2^ = 23%) following treatment. Isotretinoin was not associated with the risk of all psychiatric disorders (RR, 1.08; 95% CI, 0.99-1.19; *I*^2^ = 0%). Study-level meta-regression found that studies with participants of older age reported lower 1-year absolute risk of depression, while those with a higher percentage of male participants reported a higher 1-year absolute risk of completed suicide.

**Conclusions and Relevance:**

The findings suggest that at a population level, isotretinoin users do not have increased risk of suicide or psychiatric conditions but may instead have a lower risk of suicide attempts at 2 to 4 years following treatment. While these findings are reassuring, clinicians should continue to practice holistic psychodermatologic care and monitor patients for signs of mental distress during isotretinoin treatment.

## Key Points

**Question:**

Is isotretinoin use associated with the risk of suicide and psychiatric disorders?

**Findings:**

In this meta-analysis of 25 studies including 1 625 891 participants, the 1-year absolute risk of completed suicide, suicide attempt, suicide ideation, and self-harm among isotretinoin users was less than 0.5% each, while that of depression was 3.83%. Isotretinoin was not associated with the relative risk of all psychiatric disorders, and isotretinoin users were less likely than nonusers to attempt suicide at 2 to 4 years following treatment.

**Meaning:**

These findings indicate that there is no epidemiological evidence to suggest an increased relative risk of suicide or psychiatric conditions among isotretinoin users at a population level.

## Teaser

This meta-analysis clarifies the risk of suicide and psychiatric disorders among isotretinoin users.

## Introduction

Isotretinoin is commonly prescribed for the management of severe acne vulgaris.^1^ Despite its efficacy in treating acne, isotretinoin has reportedly been associated with suicide and a range of psychiatric disorders, including depression and anxiety,^2^ which has resulted in a black box warning for suicide, depression, aggression, and psychosis issued by the US Food and Drug Administration in 2005.^3^ It is hypothesized that isotretinoin may contribute to the development of psychiatric disorders by altering the levels of neurotransmitters involved in mood regulation, such as dopamine,^4^ serotonin,^5^ and norepinephrine.^6^

The potential link between isotretinoin and psychiatric disorders has been the subject of considerable debate, with conflicting findings in the literature. While some studies have suggested that isotretinoin use may be linked to suicide and psychiatric disorders,^7,8^ others have found no such association.^9,10^ Two existing meta-analyses reported that isotretinoin use may lead to improvements in depression symptoms.^11,12^ However, these studies did not explore other psychiatric disorders previously associated with isotretinoin.

Given the widespread use of isotretinoin and the potential deleterious outcomes for mental health, it is important to clarify the association between isotretinoin and psychiatric disorders. This study aims to provide a comprehensive and up-to-date assessment of the absolute risk, relative risk, and risk factors for suicide and psychiatric disorders among isotretinoin users with the hope of providing valuable insight into the potential risks associated with isotretinoin use and guiding clinical practice in the management of acne vulgaris.

## Methods

This meta-analysis follows an a priori systematic review protocol registered with PROSPERO (CRD42023388463) and is reported in accordance with the Preferred Reporting Items for Systematic Reviews and Meta-analyses (PRISMA) guidelines.^13^ The PRISMA checklist and Meta-analysis of Observational Studies in Epidemiology (MOOSE) checklist^14^ are provided, respectively, in eTables 1 and 2 in Supplement 1. As this is a meta-analysis of published data, institutional review board approval and informed consent were not required.

### Data Sources and Search Strategy

We searched 4 databases (PubMed, Embase, Web of Science, and Scopus) from inception until January 24, 2023, using the following free-text search strategy: (isotretinoin OR Accutane OR Roaccutane OR 13-cis-retinoic acid) AND (psychiatric OR psychotic OR mood OR “mental health” OR depression OR anxiety OR bipolar OR manic OR suicide OR suicidal OR self-harm). We also hand searched the bibliographies of included articles and relevant reviews or journals.

### Study Selection and Data Extraction

Two authors (A.T. and N.C.Y.L.M.) independently screened for eligible studies using Rayyan, a web-based collaborative systematic review platform.^15^ Studies were screened based on title and abstract, followed by full-text evaluation. We included randomized clinical trials and observational studies that reported the absolute risk and risk factors for psychiatric disorders and suicide among patients with acne taking oral isotretinoin, and the relative risk of these disorders among patients taking oral isotretinoin compared with control participants not treated with isotretinoin. We accepted conference abstracts and other gray literature fulfilling these criteria. We excluded letters, reviews, case reports, and studies published in languages other than English. Using a standardized extraction sheet, 2 authors (A.T. and N.C.Y.L.M.) extracted data comprising first author, year published, study design, study setting, country, sample size, percentage of male participants, median or mean age, intervention or exposure, outcomes, covariates, statistical methods, and key findings. Data on race and ethnicity were not consistently available and were not collected.

### Quality of Evidence

We evaluated the risk of bias at the study level using the Newcastle-Ottawa Scale (NOS) since all included studies were observational (eTable 3 in Supplement 1).^16,17^ In accordance with past reviews, we graded studies as having a high (<5 stars), moderate (5-7 stars), or low (≥8 stars) risk of bias.^18,19^ We used the Grading of Recommendations, Assessment, Development, and Evaluations system to evaluate the quality of pooled evidence at the outcome level (eTable 4 in Supplement 1).^20^

### Statistical Analysis

We found sufficient data to meta-analyze the absolute and relative risk of suicide and psychiatric disorders in isotretinoin users. Using the generic inverse variance method, we separately pooled the absolute risk and relative risk ratios (RRs) for suicide and psychiatric disorders. For the analysis on relative risk, we favored maximally covariate-adjusted estimates where available. We included 1 study that reported odds ratios (ORs),^21^ 2 studies that reported hazard ratios,^10,22^ and 2 studies that reported standardized incidence ratios,^9,23^ as these sufficiently approximate RRs.^24,25^ We used random-effects models to account for the anticipated heterogeneity and evaluated between-study heterogeneity with the *I*^2^ statistic.^26^ For outcomes with significant heterogeneity, we conducted predetermined, exploratory meta-regression of the following study-level characteristics: average age, percentage of male participants, duration of follow-up, study design (prospective or retrospective), and risk of bias (using NOS). There were insufficient studies to perform meaningful subgroup analyses. We assessed publication bias via visual inspection of funnel plot asymmetry, Egger bias, or trim-and-fill method, as appropriate. We conducted all analyses using RStudio, version 2022.07.2 (Posit Software, PBC) in accordance with statistical approaches laid out in the *Cochrane Handbook for Systematic Reviews of Interventions*.^17^ We considered a 2-sided *P* < .05 as statistically significant for the purpose of these analyses.

## Results

**Figure 1.**
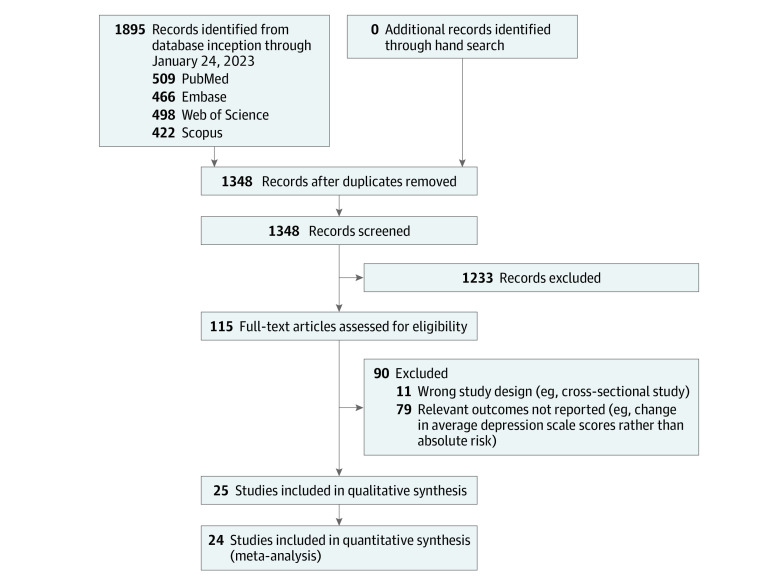
PRISMA Flow Diagram of Study Selection

The PRISMA flow diagram is shown in Figure 1. The systematic search retrieved 1895 results, and 547 duplicates were subsequently removed. Records were screened by title and abstract, with 1233 articles excluded as they did not report relevant outcomes or were inappropriate study types. Further screening by full text excluded 90 articles. The review comprised 25 articles,^9,10,21,22,23,27,28,29,30,31,32,33,34,35,36,37,38,39,40,41,42,43,44,45,46^ of which 24 were included in the meta-analysis.^9,10,21,22,23,27,28,29,30,31,32,33,34,35,36,37,38,39,40,41,42,43,44,45^

### Study Characteristics

All 25 studies were observational, with 10 prospective cohorts,^28,30,31,32,35,36,37,38,40,43^ 13 retrospective cohorts,^9,10,21,22,23,29,33,34,39,41,42,44,45^ 1 case crossover study,^27^ and 1 case-control study.^46^ Mean participant age ranged from 16 to 38 years. Three studies were conducted in Asia,^10,38,43^ 1 in Australasia,^39^ 10 in Europe,^9,28,29,32,34,35,36,37,40,45^ and 9 in North America.^21,27,30,31,33,34,41,42,44^ Three studies comprised multinational cohorts.^21,22,34^ All studies used *International Classification of Diseases* codes and relevant scales to define psychiatric events. Using the NOS, 3 studies had a high risk of bias,^29,33,41^ 16 had a moderate risk of bias,^9,21,22,28,31,32,35,36,37,38,39,40,42,43,44,45^ and 6 had a low risk of bias^9,10,23,27,30,34^ (eTable 3 in Supplement 1). A summary of the included studies is shown in eTable 5A-C in Supplement 1.

### Meta-Analysis of 1-Year Absolute Risk of Psychiatric Disorders

**Figure 2.**
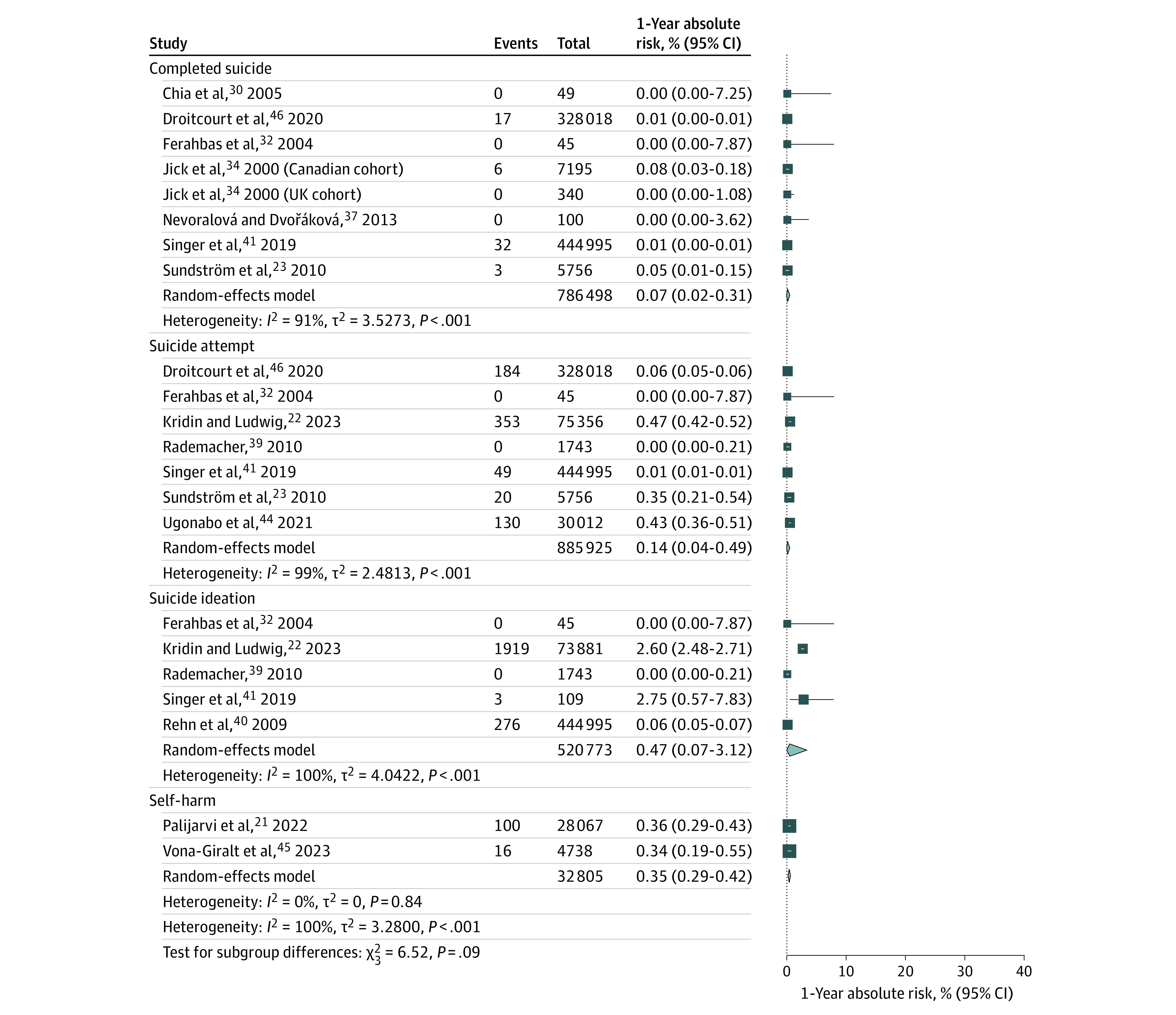
Random-Effects Meta-Analyses of the 1-Year Absolute Risk of Completed Suicide, Suicide Attempt, Suicide Ideation, and Self-Harm Diamonds represent the estimated pooled absolute risk for each meta-analysis; gray box sizes, the relative weight apportioned to studies in each meta-analysis.

**Figure 3.**
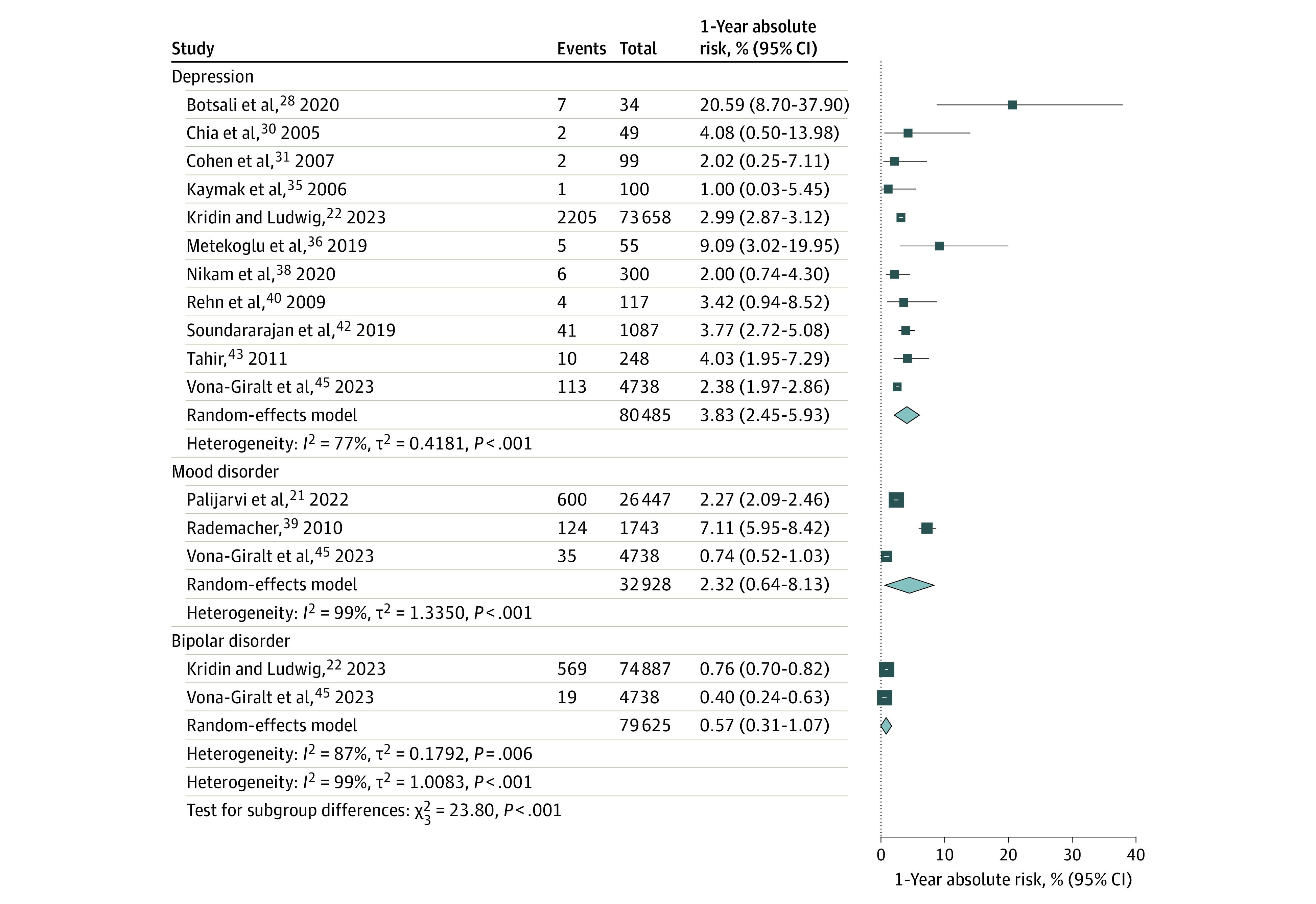
Random-Effects Meta-Analyses of the 1-Year Absolute Risk of Depression, Mood Disorder, and Bipolar Disorder Diamonds represent the estimated pooled absolute risk for each meta-analysis; gray box sizes, the relative weight apportioned to studies in each meta-analysis.

The 1-year absolute risk of completed suicide was 0.07% (95% CI, 0.02-0.31; *I*^2^ = 91%; 7 studies with 8 cohorts including 786 498 participants)^9,23,30,32,34,37,41^; of suicide attempt, 0.14% (95% CI, 0.04-0.49; *I*^2^ = 99%; 7 studies including 885 925 participants)^9,22,23,32,39,41,44^; of suicide ideation, 0.47% (95% CI, 0.07-3.12; *I*^2^ = 100%; 5 studies including 520 773 participants)^22,32,39,40,41^; and of self-harm, 0.35% (95% CI, 0.29-0.42; *I*^2^ = 0%; 2 studies including 32 805 participants)^21,45^ (Figure 2). As seen in Figure 3, the 1-year absolute risk of depression was 3.83% (95% CI, 2.45-5.93; *I*^2^ = 77%; 11 studies including 80 485 participants)^22,28,30,31,35,36,38,40,42,43,45^; of mood disorder, 2.32% (95% CI, 0.64-8.13; *I*^2^ = 99%; 3 studies including 32 928 participants)^21,39,45^; and of bipolar disorder, 0.57% (95% CI, 0.31-1.07; *I*^2^ = 87%; 2 studies including 79 625 participants).^22,45^ The 1-year absolute risk of all psychiatric disorders was 4.57% (95% CI, 1.58-12.48; *I*^2^ = 100%; 4 studies with 5 cohorts including 61 850 participants)^21,34,44,45^; of anxiety, 6.67% (95% CI, 2.77-15.19; *I*^2^ = 100%; 4 studies including 96 196 participants)^21,22,28,45^; of psychotic disorders, 0.13% (95% CI, 0.08-0.23; *I*^2^ = 77%; 3 studies including 108 698 participants)^21,22,45^; and of sleep disorders, 0.74% (95% CI, 0.27-1.98; *I*^2^ = 99%; 5 studies including 252 763 participants)^21,33,39,43,45^ (eFigure 1 in Supplement 1).

#### Meta-Regression

The meta-analysis for 1-year absolute risk of depression comprised sufficient studies for meta-regression of prespecified study-level characteristics. Average age was identified as a significant effect moderator, accounting for 41.9% of heterogeneity (eFigure 2 and eTable 6 in Supplement 1). The pooled 1-year absolute risk of depression decreased by a factor of 0.15 (95% CI, −0.03 to −0.28) per year increase in age. Other characteristics, including sex, follow-up duration, study design, and NOS score, were not significant effect moderators. Meta-regression for 1-year absolute risk of completed suicide identified sex and study design as significant effect moderators, accounting for 38.6% and 59.3% of heterogeneity, respectively (eFigures 3 and 4 and eTable 7 in Supplement 1). The pooled 1-year absolute risk of completed suicide increased by a factor of 0.16 (95% CI, 0.01-0.32) for every percentage increase in male sex in the study cohort. The pooled 1-year absolute risk of completed suicide was lower in retrospective studies than prospective studies (−3.57; 95% CI, −5.97 to −1.16). Follow-up duration, average age, and NOS score were not significant effect moderators.

#### Publication Bias

Visual inspection suggested possible funnel plot asymmetry, and the trim-and-fill method imputed 5 missing studies for 1-year absolute risk of completed suicide, 2 for suicide attempt, 1 and 2 for all psychiatric disorders with minimal change to the pooled absolute risk (eFigure 5 in Supplement 1). Visual inspection suggested no funnel plot asymmetry, and the trim-and-fill method imputed no missing studies for sleep disorders. For depression, visual inspection suggested possible funnel plot asymmetry, and the trim-and-fill method imputed 1 missing study, with minimal change to the pooled absolute risk (3.11%; 95% CI, 1.74-5.50; *I*^2^ = 84%) (12 studies).

### Absolute Risk of Psychiatric Disorders at 5 Years and 10 Years

One study reported the 5-year absolute risk of all psychiatric disorders (25.16%; 95% CI, 23.74%-26.63%), suicide ideation (0.03%; 95% CI, 0.00%-0.16%), sleep disorders (0.54%; 95% CI, 0.32%-0.84%), and mood disorders (6.67%; 95% CI, 5.87%-7.54%).^29^ Another study reported the 10-year absolute risk of all psychiatric disorders (38.29%; 95% CI, 37.34%-39.25%), depression (1.31%; 95% CI, 1.10%-1.56%), anxiety (10.47%; 95% CI, 9.88%-11.09%), completed suicide (3.02%; 95% CI, 2.69%-3.37%), psychotic disorders (0.03%; 95% CI, 0.01%-0.09%), and bipolar disorder (0.10%; 95% CI, 0.05%-0.18%).^10^

### Meta-Analysis on Relative Risk of Psychiatric Disorders

**Figure 4.**
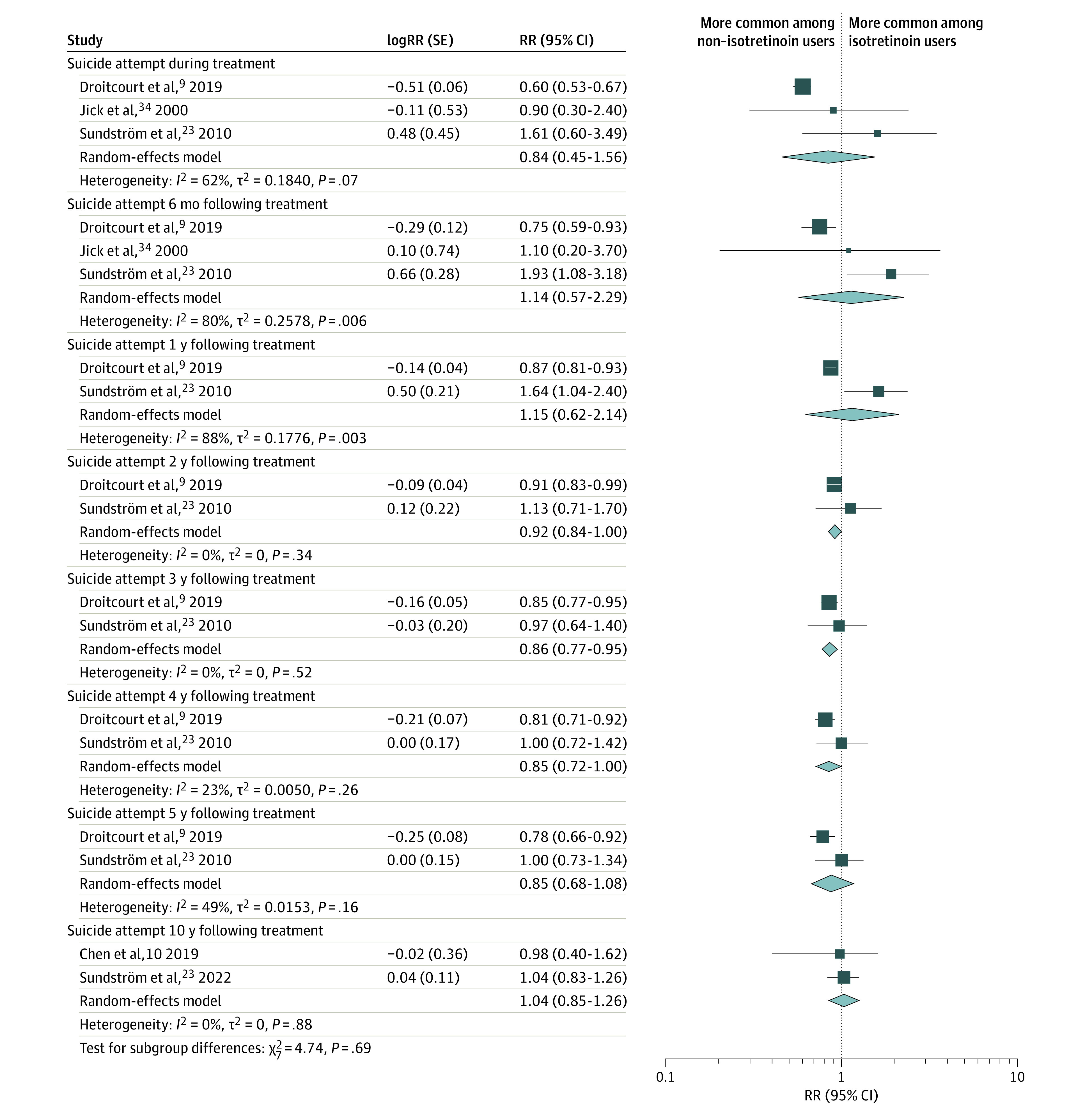
Random-Effects Meta-Analyses of the Association Between Isotretinoin Use and Relative Risk of Suicide Attempt During Treatment and at 6 Months and 1, 2, 3, 4, 5, and 10 Years Following Treatment Diamonds represent the estimated pooled relative risk ratio (RR) for each meta-analysis; gray box sizes, the relative weight apportioned to studies in each meta-analysis.

The pooled associations of isotretinoin use with suicide are shown in Figure 4. After adjustment for age, participants taking isotretinoin were less likely than nonusers to attempt suicide at 2 years (RR, 0.92; 95% CI, 0.84-1.00; *I*^2^ = 0%; 2 studies including 449 570 participants),^9,23^ 3 years (RR, 0.86; 95% CI, 0.77-0.95; *I*^2^ = 0%; 2 studies including 449 570 participants),^9,23^ and 4 years (RR, 0.85; 95% CI, 0.72-1.00; *I*^2^ = 23%; 2 studies including 449 570 participants)^9,23^ following treatment. There was no association between isotretinoin use and suicide attempt during treatment (RR, 0.84; 95% CI, 0.45-1.56; *I*^2^ = 62%; 3 studies including 456 765 participants)^9,23,34^ and at 6 months (RR, 1.14; 95% CI, 0.57-2.29; *I*^2^ = 80%; 3 studies including 456 765 participants),^9,23,34^ 1 year (RR, 1.15; 95% CI, 0.62-2.14; *I*^2^ = 88%; 2 studies including 449 570 participants),^9,23^ 5 years (RR, 0.85; 95% CI, 0.68-1.08; *I*^2^ = 49%; 2 studies including 449 570 participants),^9,23^ and 10 years (RR, 1.04; 95% CI, 0.85-1.26; *I*^2^ = 0%; 2 studies including 35 699 participants)^10,23^ following treatment.

Isotretinoin users were not at higher risk of all psychiatric disorders (RR, 1.08; 95% CI, 0.99-1.19; *I*^2^ = 0%; 4 studies including 59 247 participants),^21,34,45^ depression (RR, 1.46; 95% CI, 0.55-3.87; *I*^2^ = 80%; 2 studies including 73 784 participants),^22,27^ anxiety (RR, 0.97; 95% CI, 0.73-1.30; *I*^2^ = 97%; 2 studies including 117 402 participants),^21,22^ psychotic disorders (RR, 0.80; 95% CI, 0.41-1.58; *I*^2^ = 78%; 2 studies including 132 324 participants),^21,22^ and sleep disorders (RR, 1.61; 95% CI, 0.89-2.93; *I*^2^ = 98%; 2 studies including 273 541 participants)^21,33^ at 1 year following treatment (eFigure 6 in Supplement 1). There were insufficient studies to perform a subgroup analysis and assess publication bias.

### Relative Risk of Psychiatric Disorders at 10 Years

One study reported the hazards of all psychiatric disorders, depression, anxiety, and psychotic disorder at 10 years following treatment.^10^ The hazard ratios were 1.009 (95% CI, 0.422-1.696) for all psychiatric disorders, 0.953 (95% CI, 0.398-1.613) for depression, 1.022 (95% CI, 0.428-1.711) for anxiety, and 1.000 (95% CI, 0.418-1.692) for psychotic disorders.

### Risk Factors for Psychiatric Disorders

Three studies investigated the risk factors for psychiatric disorders among isotretinoin users. Vona-Giralt et al^45^ reported that isotretinoin users with a psychiatric history were more likely to develop incident psychiatric disorders (incidence rate ratio, 1.60; 95% CI, 1.34-1.92). Droitcourt et al^46^ found that the cumulative dose of isotretinoin (in increments of 1000 mg) was associated with a lower risk of suicide attempt (OR, 0.77; 95% CI, 0.68-0.89), while patients with a psychiatric history were more likely to attempt suicide (OR, 18.21; 95% CI, 9.96-33.30).^46^ Specifically, a previous diagnosis of anxiety was associated with a higher risk of suicide attempt (OR, 4.78; 95% CI, 2.44-9.33). Daily dose of isotretinoin (in increments of 20 mg), isotretinoin brand name, and type of prescriber (dermatologist vs general practitioner) were not associated with suicide attempt. According to Chen et al,^10^ there was no increase in risk of psychiatric disorders in patients who received a different daily dose or duration of isotretinoin treatment.

## Discussion

This meta-analysis of 24 studies including 1 625 891 participants suggests a low absolute risk and no increased relative risk of suicide and psychiatric disorders among patients taking isotretinoin. In fact, our findings suggest that isotretinoin may be associated with a lower risk of suicide attempt at 2 to 4 years following treatment. Having a psychiatric history was associated with an increased risk of suicide attempt and psychiatric disorders among isotretinoin users, while a higher cumulative dose of isotretinoin was associated with a lower risk of suicide attempt. The meta-regression showed that studies with older average age of the study population reported a lower 1-year absolute risk of depression. Studies with a higher percentage of male participants were more likely to report incident completed suicide, which is consistent with the sex distribution of suicide shown in the existing literature.^47^

Our meta-analysis shows that 4.57% of isotretinoin users developed a psychiatric disorder over a 1-year period. The 1-year absolute risk of self-harm, suicide ideation, suicide attempt, and completed suicide were each less than 0.5%. The pooled 1-year absolute risk of suicide attempt was 0.14%, which is lower than the 1-year absolute risk of suicide attempts in adolescents reported in 2 longitudinal studies (0.84% and 1.3%, respectively).^48,49^ The pooled 1-year absolute risk of depression in our study was 3.83%, which is comparable with the absolute risk of depression in adolescents reported in 2 previous studies (3.3% and 5.72%, respectively).^50,51^ While our findings are reassuring, clinicians should remain vigilant in monitoring patients for signs of psychiatric distress during isotretinoin treatment.

The relationship among acne, isotretinoin, and psychiatric disorders is a complex one. Prior challenge-dechallenge-rechallenge studies have provided strong evidence for a direct causal relationship between isotretinoin use and mood changes in rare individuals, via biological effects on the central nervous system.^3,7,8^ This may be an idiosyncratic reaction that is difficult to predict.^11^ However, there may be a second indirect effect of isotretinoin on improved mood, mediated by improved acne and self-image^11,52^; this is consistent with our meta-analysis, which shows no increased epidemiological risk of suicide or depression among isotretinoin users. Hence, while clinicians should remain vigilant and provide counseling for rare idiosyncratic mood changes that could increase the risk of suicide, they should be aware that isotretinoin appears to be safe at a population level.

The study of isotretinoin and mood changes may be influenced by various biases. First, the observational studies included in this meta-analysis are subject to confounding by indication, where the indication for selecting a particular treatment also affects the outcome of interest.^53^ In view of the prominence given to claims of suicide and psychiatric adverse effects of isotretinoin among physicians and the media over the years, it is plausible that patients deemed to be at higher risk of psychiatric illness were less likely to receive isotretinoin,^9^ which may have resulted in an underestimation of the psychiatric risks of isotretinoin in these observational studies. Second, studies examining psychiatric disorders in isotretinoin users may have been subject to detection bias. Patients taking isotretinoin may be more closely monitored for mood changes, which could result in increased detection of psychiatric outcomes; however, our meta-analysis did not detect any increased risk of psychiatric disorders among isotretinoin users. Detection bias may also act in the opposite direction, as early detection of psychiatric disorders allows for timely treatment, which in turn may lower the risk of suicide.^54^

At present, few studies have explored the risk factors associated with suicide and psychiatric disorders among isotretinoin users, indicating a need for more research. The insights from future studies may guide clinicians in balancing the benefits and potential risks of prescribing isotretinoin treatment with improved patient outcomes.

### Strengths and Limitations

The strengths of our meta-analysis lie in the large number of systematically included studies comprising a combined cohort of diverse backgrounds, adding to the generalizability of the study findings. Nonetheless, our findings should be interpreted in due consideration of the following limitations.

First, our findings are limited by substantial heterogeneity, and we were able to perform meta-regression for only 2 outcomes to investigate the sources of heterogeneity. However, such heterogeneity may not be clinically significant, as the *I*^2^ statistic is known to increase rapidly with larger sample sizes of included studies.^55^ Second, meta-analyses for various outcomes were limited by imprecision. For example, estimates for relative risk of depression and suicide attempt had wide CIs, suggesting that these analyses were relatively underpowered or heterogenous. While our findings are largely reassuring, we are unable to exclude the potential for meaningful increased risks for the outcomes with wide CIs. Third, the majority of studies included in the analysis for relative risk only adjusted for age and sex without accounting for other potential confounders such as severity of acne, medical comorbidities, and socioeconomic status. Fourth, the included database studies did not specifically assess for psychiatric disorders in all isotretinoin users; thus, there may be underdiagnosis of incident psychiatric disorders. Fifth, few studies were included in the meta-analyses for relative risk of psychiatric disorders due to limitations in the existing literature. Finally, all studies were subject to confounding by indication and detection bias, both of which could bias our findings either way, and it is not possible to measure this effect.

## Conclusions

Pooled epidemiological evidence from this meta-analysis, though limited by heterogeneity and imprecision, suggests that there is no increased risk of suicide or psychiatric conditions among isotretinoin users at a population level. In fact, isotretinoin use was associated with a lower risk of suicide attempt at 2 to 4 years following treatment. Further research is needed to identify risk factors for psychiatric disorders among isotretinoin users. While our findings are reassuring, clinicians should continue to practice holistic psychodermatologic care and monitor patients for signs of mental distress during isotretinoin treatment.

## Notes

Supplement 1. **eTable 1.** PRISMA Checklist **eTable 2.** MOOSE Checklist **eTable 3.** Evaluation of Risk of Bias Using Newcastle-Ottawa Scale **eTable 4.** Evaluation of Quality of Pooled Evidence Using the Grading of Recommendations, Assessment, Development, and Evaluations (GRADE) Framework **eTable 5**A. Summary of Included Studies for Cumulative Incidence of Psychiatric Disorders **eTable 5**B. Summary of Included Studies for Relative Risk of Psychiatric Disorders **eTable 5**C. Summary of Included Studies for Predictors of Psychiatric Disorders **eTable 6.** Meta-Regression of Prespecified Study-Level Characteristics for 1-Year Incidence of Depression **eTable 7.** Meta-Regression of Prespecified Study-Level Characteristics for 1-Year Incidence of Completed Suicide **eFigure 1.** Meta-Analysis for 1-Year Incidence of All Psychiatric Disorders, Anxiety, Psychotic Disorders, and Sleep Disorders **eFigure 2.** Meta-Regression for 1-Year Incidence of Depression (Average Age) **eFigure 3.** Meta-Regression for 1-Year Incidence of Completed Suicide (Sex) **eFigure 4.** Meta-Regression for 1-Year Incidence of Completed Suicide (Study Design) **eFigure 5.** Contour-Enhanced Funnel Plots, With Missing Studies Imputed Via the Trim-and-Fill Method **eFigure 6.** Meta-Analysis for Relative Risk of All Psychiatric Disorders, Depression, Anxiety, Psychotic Disorders, and Sleep Disorders
eReferences

Supplement 2.
Data Sharing Statement
