# Supplementary material for: Risk of Suicide and Psychiatric Disorders Among Isotretinoin Users: A Meta-Analysis
Source: JAMA Dermatol. 2023 Nov 29;160(1):54–62. doi: 10.1001/jamadermatol.2023.4579 (PMC10687715; doi:10.1001/jamadermatol.2023.4579)
Supplement: Supplement 1. — eTable 1. PRISMA Checklist eTable 2. MOOSE Checklist eTable 3. Evaluation of Risk of Bias Using Newcastle-Ottawa Scale eTable 4. Evaluation of Quality of Pooled Evidence Using the Grading of Recommendations, Assessment, Development, and Evaluations (GRADE) Framework eTable 5A. Summary of Included Studies for Cumulative Incidence of Psychiatric Disorders eTable 5B. Summary of Included Studies for Relative Risk of Psychiatric Disorders eTable 5C. Summary of Included Studies for Predictors of Psychiatric Disorders eTable 6. Meta-Regression of Prespecified Study-Level Characteristics for 1-Year Incidence of Depression eTable 7. Meta-Regression of Prespecified Study-Level Characteristics for 1-Year Incidence of Completed Suicide eFigure 1. Meta-Analysis for 1-Year Incidence of All Psychiatric Disorders, Anxiety, Psychotic Disorders, and Sleep Disorders eFigure 2. Meta-Regression for 1-Year Incidence of Depression (Average Age) eFigure 3. Meta-Regression for 1-Year Incidence of Completed Suicide (Sex) eFigure 4. Meta-Regression for 1-Year Incidence of Completed Suicide (Study Design) eFigure 5. Contour-Enhanced Funnel Plots, With Missing Studies Imputed Via the Trim-and-Fill Method eFigure 6. Meta-Analysis for Relative Risk of All Psychiatric Disorders, Depression, Anxiety, Psychotic Disorders, and Sleep Disorders eReferences [file jamadermatol-e234579-s001.pdf]

Supplemental Online Content

Tan NKW, Tang A, MacAlevey NCYL, Tan BKJ, Oon HH. Risk of suicide and psychiatric disorders among isotretinoin users: a meta-analysis. *JAMA Dermatol*. Published online November 29, 2023. doi:10.1001/jamadermatol.2023.4579

eTable 1. PRISMA Checklist

eTable 2. MOOSE Checklist

eTable 3. Evaluation of Risk of Bias Using Newcastle-Ottawa Scale

eTable 4. Evaluation of Quality of Pooled Evidence Using the Grading of Recommendations Assessment, Development, and Evaluations (GRADE) Framework

eTable 5A. Summary of Included Studies for Absolute Risk of Psychiatric Disorders

eTable 5B. Summary of Included Studies for Relative Risk of Psychiatric Disorders

eTable 5C. Summary of Included Studies for Risk Factors of Psychiatric Disorders

eTable 6. Meta-Regression of Prespecified Study-Level Characteristics for 1-Year Absolute Risk of Depression

eTable 7. Meta-Regression of Prespecified Study-Level Characteristics for 1-Year Absolute Risk of Completed Suicide

eFigure 1. Meta-Analysis for 1-Year Absolute Risk of All Psychiatric Disorders, Anxiety, Psychotic Disorders, and Sleep Disorders

eFigure 2. Meta-Regression for 1-Year Absolute Risk of Depression (Average Age)

eFigure 3. Meta-Regression for 1-Year Absolute Risk of Completed Suicide (Sex)

eFigure 4. Meta-Regression for 1-Year Absolute Risk of Completed Suicide (Study Design)

eFigure 5. Contour-Enhanced Funnel Plots, With Missing Studies Imputed Via the Trim-and-Fill Method

eFigure 6. Meta-Analysis for Relative Risk of All Psychiatric Disorders, Depression, Anxiety, Psychotic Disorders, and Sleep Disorders

eReferences

This supplemental material has been provided by the authors to give readers additional information about their work.

© 2023 Tan NKW et al. *JAMA Dermatology*.

eTable 1. PRISMA Checklist[1]

Section and Topic	Item #	Checklist item	Reported on page #
**TITLE**			
Title	1	Identify the report as a systematic review.	1
**ABSTRACT**			
Abstract	2	See the PRISMA 2020 for Abstracts checklist.	3-4
**INTRODUCTION**			
Rationale	3	Describe the rationale for the review in the context of existing knowledge.	5
Objectives	4	Provide an explicit statement of the objective(s) or question(s) the review addresses.	5
**METHODS**			
Eligibility criteria	5	Specify the inclusion and exclusion criteria for the review and how studies were grouped for the syntheses.	7-8
Information sources	6	Specify all databases, registers, websites, organisations, reference lists and other sources searched or consulted to identify studies. Specify the date when each source was last searched or consulted.	7
Search strategy	7	Present the full search strategies for all databases, registers and websites, including any filters and limits used.	7
Selection process	8	Specify the methods used to decide whether a study met the inclusion criteria of the review, including how many reviewers screened each record and each report retrieved, whether they worked independently, and if applicable, details of automation tools used in the process.	7-8
Data collection process	9	Specify the methods used to collect data from reports, including how many reviewers collected data from each report, whether they worked independently, any processes for obtaining or confirming data from study investigators, and if applicable, details of automation tools used in the process.	8
Data items	10a	List and define all outcomes for which data were sought. Specify whether all results that were compatible with each outcome domain in each study were sought (e.g. for all measures, time points, analyses), and if not, the methods used to decide which results to collect.	8-9
	10b	List and define all other variables for which data were sought (e.g. participant and intervention characteristics, funding sources). Describe any assumptions made about any missing or unclear information.	8-9
Study risk of bias assessment	11	Specify the methods used to assess risk of bias in the included studies, including details of the tool(s) used, how many reviewers assessed each study and whether they worked independently, and if applicable, details of automation tools used in the process.	8
Effect measures	12	Specify for each outcome the effect measure(s) (e.g. risk ratio, mean difference) used in the synthesis or presentation of results.	8-9

© 2023 Tan NKW et al. *JAMA Dermatology*.

Section and Topic	Item #	Checklist item	Reported on page #
Synthesis methods	13a	Describe the processes used to decide which studies were eligible for each synthesis (e.g. tabulating the study intervention characteristics and comparing against the planned groups for each synthesis (item #5)).	8-9
	13b	Describe any methods required to prepare the data for presentation or synthesis, such as handling of missing summary statistics, or data conversions.	8-9
	13c	Describe any methods used to tabulate or visually display results of individual studies and syntheses.	9
	13d	Describe any methods used to synthesize results and provide a rationale for the choice(s). If meta-analysis was performed, describe the model(s), method(s) to identify the presence and extent of statistical heterogeneity, and software package(s) used.	9
	13e	Describe any methods used to explore possible causes of heterogeneity among study results (e.g. subgroup analysis, meta-regression).	9
	13f	Describe any sensitivity analyses conducted to assess robustness of the synthesized results.	9
Reporting bias assessment	14	Describe any methods used to assess risk of bias due to missing results in a synthesis (arising from reporting biases).	8-9
Certainty assessment	15	Describe any methods used to assess certainty (or confidence) in the body of evidence for an outcome.	9
**RESULTS**			
Study selection	16a	Describe the results of the search and selection process, from the number of records identified in the search to the number of studies included in the review, ideally using a flow diagram.	10, Figure 1
	16b	Cite studies that might appear to meet the inclusion criteria, but which were excluded, and explain why they were excluded.	Figure 1
Study characteristics	17	Cite each included study and present its characteristics.	10, eTable 5a-c
Risk of bias in studies	18	Present assessments of risk of bias for each included study.	10, eTable 3
Results of individual studies	19	For all outcomes, present, for each study: (a) summary statistics for each group (where appropriate) and (b) an effect estimate and its precision (e.g. confidence/credible interval), ideally using structured tables or plots.	Figure 2-4
Results of syntheses	20a	For each synthesis, briefly summarise the characteristics and risk of bias among contributing studies.	eTable 3, eTable 5a-c
	20b	Present results of all statistical syntheses conducted. If meta-analysis was done, present for each the summary estimate and its precision (e.g. confidence/credible interval) and measures of statistical heterogeneity. If comparing groups, describe	10-13, Figure 2-4, eFigure

© 2023 Tan NKW et al. *JAMA Dermatology*.

Section and Topic	Item #	Checklist item	Reported on page #
		the direction of the effect.	1, 7
	20c	Present results of all investigations of possible causes of heterogeneity among study results.	11, eFigure 2, eTable 6, eFigure 3-4, eTable 7
	20d	Present results of all sensitivity analyses conducted to assess the robustness of the synthesized results.	11, eFigure 2, eTable 6, eFigure 3-4, eTable 7
Reporting biases	21	Present assessments of risk of bias due to missing results (arising from reporting biases) for each synthesis assessed.	12, eFigure 5
Certainty of evidence	22	Present assessments of certainty (or confidence) in the body of evidence for each outcome assessed.	eTable 4
**DISCUSSION**			
Discussion	23a	Provide a general interpretation of the results in the context of other evidence.	15-18
	23b	Discuss any limitations of the evidence included in the review.	19
	23c	Discuss any limitations of the review processes used.	19
	23d	Discuss implications of the results for practice, policy, and future research.	15-19
**OTHER INFORMATION**			
Registration and protocol	24a	Provide registration information for the review, including register name and registration number, or state that the review was not registered.	7
	24b	Indicate where the review protocol can be accessed, or state that a protocol was not prepared.	7
	24c	Describe and explain any amendments to information provided at registration or in the protocol.	-
Support	25	Describe sources of financial or non-financial support for the review, and the role of the funders or sponsors in the review.	19
Competing interests	26	Declare any competing interests of review authors.	19
Availability of data, code and other materials	27	Report which of the following are publicly available and where they can be found: template data collection forms; data extracted from included studies; data used for all analyses; analytic code; any other materials used in the review.	-

© 2023 Tan NKW et al. *JAMA Dermatology*.

eTable 2. MOOSE Checklist[2]

Item No	Recommendation	Reported on Page No
Reporting of background should include		
1	Problem definition	5
2	Hypothesis statement	5
3	Description of study outcome(s)	5
4	Type of exposure or intervention used	5
5	Type of study designs used	5
6	Study population	5
Reporting of search strategy should include		
7	Qualifications of searchers (eg, librarians and investigators)	1
8	Search strategy, including time period included in the synthesis and key words	7
9	Effort to include all available studies, including contact with authors	7-8
10	Databases and registries searched	7
11	Search software used, name and version, including special features used (eg, explosion)	7
12	Use of hand searching (eg, reference lists of obtained articles)	7
13	List of citations located and those excluded, including justification	7-8, Figure 1
14	Method of addressing articles published in languages other than English	8
15	Method of handling abstracts and unpublished studies	8
16	Description of any contact with authors	-
Reporting of methods should include		
17	Description of relevance or appropriateness of studies assembled for assessing the hypothesis to be tested	7-8
18	Rationale for the selection and coding of data (eg, sound clinical principles or convenience)	7-8

© 2023 Tan NKW et al. *JAMA Dermatology*.

Item No	Recommendation	Reported on Page No
19	Documentation of how data were classified and coded (eg, multiple raters, blinding and interrater reliability)	8
20	Assessment of confounding (eg, comparability of cases and controls in studies where appropriate)	8
21	Assessment of study quality, including blinding of quality assessors, stratification or regression on possible predictors of study results	8
22	Assessment of heterogeneity	8-9
23	Description of statistical methods (eg, complete description of fixed or random effects models, justification of whether the chosen models account for predictors of study results, dose-response models, or cumulative meta-analysis) in sufficient detail to be replicated	8-9
24	Provision of appropriate tables and graphics	Fig 1, eTable 5a-c
Reporting of results should include		
25	Graphic summarizing individual study estimates and overall estimate	Figure 2-4, eFigure 1,6
26	Table giving descriptive information for each study included	eTable 5a-c
27	Results of sensitivity testing (eg, subgroup analysis)	11, eFigure 2, eTable 6, eFigure 3-4, eTable 7
28	Indication of statistical uncertainty of findings	11, eFigure 2, eTable 6, eFigure 3-4, eTable 7
Reporting of discussion should include		
29	Quantitative assessment of bias (eg, publication bias)	12, eFigure 5
30	Justification for exclusion (eg, exclusion of non-English language citations)	7
31	Assessment of quality of included studies	10, eTable 3-4
Reporting of conclusions should include		
32	Consideration of alternative explanations for observed results	15-19

© 2023 Tan NKW et al. *JAMA Dermatology*.

Item No	Recommendation	Reported on Page No
33	Generalization of the conclusions (ie, appropriate for the data presented and within the domain of the literature review)	15-19
34	Guidelines for future research	15-19
35	Disclosure of funding source	19

© 2023 Tan NKW et al. *JAMA Dermatology*.

eTable 3. Evaluation of Risk of Bias Using Newcastle-Ottawa Scale[3,4]

Cohort study	Representativeness of exposed cohort	Selection of non-exposed cohort	Ascertainment of exposure	Demonstration that outcome of interest was not present at start of study	Adjusts for age	Adjusts for sex	Assessment of outcome	Sufficient follow-up period	Adequacy of follow-up	Total	Risk of bias*
Botsali 2020[5]	0	0	1	1	0	0	1	1	1	5	Moderate
Brzezinski 2017[6]	1	0	1	0	0	0	1	1	0	4	High
Chen 2022[7]	1	1	1	1	1	1	1	1	1	9	Low
Chia 2005[8]	1	1	1	1	0	1	1	1	1	8	Low
Cohen 2007[9]	1	1	1	0	0	0	1	1	0	5	Moderate
Droitcourt 2019[10]	1	1	1	0	1	1	1	1	0	7	Moderate
Ferahbas 2004[11]	0	0	1	1	0	0	1	1	1	5	Moderate
Gupta 2020[12]	1	1	1	0	0	0	1	0	0	4	High
Jick 2000[13]	1	1	1	1	1	1	1	1	0	8	Low
Kaymak 2006[14]	1	0	1	0	0	0	1	1	1	5	Moderate
Kridin 2022[15]	1	1	1	0	1	1	1	1	0	7	Moderate
Metekoglu 2019[16]	1	0	1	0	0	0	1	1	1	5	Moderate
Nevoralova 2013[17]	1	0	1	0	0	0	1	1	1	5	Moderate
Nikam 2020[18]	1	0	1	0	0	0	1	1	1	5	Moderate
Paljarvi 2022[19]	1	1	1	0	1	1	1	1	0	7	Moderate
Rademaker 2010[20]	1	0	1	0	0	0	1	1	1	5	Moderate
Rehn 2009[21]	1	0	1	0	0	0	1	1	1	5	Moderate
Singer 2019[22]	1	0	1	0	0	0	1	1	0	4	High
Soundararajan 2019[23]	1	1	1	0	1	1	1	1	0	7	Moderate
Sundström 2010[24]	1	1	1	0	1	1	1	1	1	8	Low
Tahir 2011[25]	1	0	1	0	0	0	1	1	1	5	Moderate
Ugonabo 2021[26]	1	1	1	0	1	1	1	1	0	7	Moderate
Vona-Giralt 2022[27]	1	1	1	0	1	0	1	1	1	7	Moderate

Case-control study	Representativeness of exposed cohort	Selection of non-exposed cohort	Ascertainment of exposure	Demonstration that outcome of interest was not present at start of study	Adjusts for age	Adjusts for sex	Assessment of outcome	Sufficient follow-up period	Adequacy of follow-up	Total	Risk of bias*
Azoulay 2008[28]	1	1		1	1	1	1	1	1	8	Low
Droitcourt 2020[29]	1	1	1	1	1	1	1	1	1	9	Low

*high (<5), moderate (5-7), low (≥8)

© 2023 Tan NKW et al. *JAMA Dermatology*.

eTable 4. Evaluation of Quality of Pooled Evidence Using the Grading of Recommendations Assessment, Development, and Evaluation (GRADE) Framework[30]

Outcomes	Pooled outcomes (95% CI) /%	No. of patients (no. of included studies)	Statistical heterogeneity	A	B	C	D	E
Absolute Risk of completed suicide	0.07 (0.02, 0.31)	786,498 (8 studies)	I^2 = 91% (*P* <0.01)	-1		-1		-1
Absolute risk of suicide attempt	0.14 (0.04, 049)	885,925 (7 studies)	I^2 = 99% (*P* <0.01)	-1		-1		
Absolute risk of suicide ideation	0.47 (0.07, 3.12)	520,773 (5 studies)	I^2 = 100% (*P* = 0)	-1		-1		
Absolute risk of self-harm	0.35 (0.29, 0.42)	32,805 (2 studies)	I^2 = 0% (*P* = 0.84)		-1			
Absolute risk of depression	3.83 (2.45, 5.93)	80,485 (11 studies)	I^2 = 77% (*P* = <0.01)			*		
Absolute risk of mood disorder	2.32 (0.64, 8.13)	32,928 (3 studies)	I^2 = 99% (*P* <0.01)	-1		-1		
Absolute risk of bipolar disorder	0.57 (0.31, 1.07)	79,625 (2 studies)	I^2 = 87% (*P* <0.01)		-1	-1		
Absolute risk of all psychiatric disorders	4.57 (1.58, 12.48)	61,850 (5 studies)	I^2 = 100% (*P* <0.01)	-1		-1		

© 2023 Tan NKW et al. *JAMA Dermatology*.

Outcomes	Pooled outcomes (95% CI) /%	No. of patients (no. of included studies)	Statistical heterogeneity	A	B	C	D	E
Absolute risk of anxiety	6.67 (2.77, 15.19)	96,196 (4 studies)	I^2 = 100% (*P* <0.01)			-1		
Absolute risk of psychotic disorder	0.13 (0.08, 0.23)	108,698 (3 studies)	I^2 = 77% (*P* = 0.01)			-1		
Absolute risk of sleep disorder	0.74 (0.27, 1.98)	252,763 (5 studies)	I^2 = 99% (*P* <0.01)	-1		-1		
Relative risk of suicide attempt – during treatment	0.84 (0.45, 1.56)	456,765 (3 studies)	I^2 = 62% (*P* =0.07)		-1	-1		
Relative risk of suicide attempt – 6 months	1.14 (0.57, 2.29)	456,765 (3 studies)	I^2 = 80% (*P* <0.01)		-1	-1		
Relative risk of suicide attempt – 1 year	1.15 (0.62, 2.14)	449,570 (2 studies)	I^2 = 88% (*P* =0.11)		-1	-1		
Relative risk of suicide attempt – 2 years	0.92 (0.84, 1.00)	449,570 (2 studies)	I^2 = 0% (*P* =0.34)		-1			
Relative risk of suicide attempt – 3 years	0.86 (0.77, 0.95)	449,570 (2 studies)	I^2 = 0% (*P* =0.52)		-1			

© 2023 Tan NKW et al. *JAMA Dermatology*.

Outcomes	Pooled outcomes (95% CI) /%	No. of patients (no. of included studies)	Statistical heterogeneity	A	B	C	D	E
Relative risk of suicide attempt – 4 years	0.85 (0.72, 1.00)	449,570 (2 studies)	I^2 = 23% (*P* =0.26)		-1			
Relative risk of suicide attempt – 5 years	0.85 (0.68, 1.08)	449,570 (2 studies)	I^2 = 49% (*P* =0.16)		-1			
Relative risk of suicide attempt – 10 years	1.04 (0.85, 1.26)	35,699 (2 studies)	I^2 = 0% (*P* =0.88)		-1			
Relative risk of all psychiatric disorders	1.08 (0.99, 1.19)	59,247 (4 studies)	I^2 = 0% (*P* =0.43)					
Relative risk of depression	1.46 (0.55, 3.87)	73,784 (2 studies)	I^2 = 80% (*P* =0.02)		-1	-1		
Relative risk of anxiety disorders	0.97 (0.73, 1.30)	117,402 (2 studies)	I^2 = 97% (*P* <0.01)		-1	-1		
Relative risk of psychotic disorder	0.80 (0.41, 1.58)	132,324 (2 studies)	I^2 = 78% (*P* =0.03)		-1	-1		
Relative risk of sleep disorder	1.61 (0.89, 2.93)	273,541 (2 studies)	I^2 = 98% (*P* <0.01)		-1	-1		

Quality of evidence for observational studies is graded starting at low quality for a causal effect, and downgraded or upgraded based on the following criteria.

© 2023 Tan NKW et al. *JAMA Dermatology*.

A: downgraded by one level for risk of bias among included studies.
B: downgraded by one level for imprecision (e.g. few studies or large confidence intervals).
C: downgraded by one level for inconsistency (e.g. moderate to substantial unexplained statistical heterogeneity with $I^2 \geq 50\%$).
D: downgraded by one level for indirectness of evidence.
E: downgraded by one level for publication bias.

*Initial detected heterogeneity was sufficiently explained by meta-regression (average age), accounting for 41.9% of heterogeneity.

© 2023 Tan NKW et al. *JAMA Dermatology*.

eTable 5A. Summary of Included Studies for Absolute Risk of Psychiatric Disorders

Study	Study design	Analytical sample size	Country	Mean age	% Male	Outcome definition	Mean follow-up duration/ years	NOS Score
Botsali 2020[5]	Prospective cohort	34	Turkey	16.1	52	Depression: Children Depression Scale (CDS)<br><br>Anxiety: State-Trait Anxiety Inventory	0.5	5
Brzezinski 2017[6]	Retrospective cohort	3525	Poland, Romania	18.15	47.63	NR	5	4
Chen 2022[7]	Retrospective cohort	9981	Taiwan	38.0	34.58	Suicidality: ICD-9-CM code E950–E959<br><br>ADHD: ICD-9-CM code 314.0–314.01<br><br>Schizophrenia: ICD-9-CM code 295–295.95<br><br>BD: ICD-9-CM code 296.4–296.7<br><br>MDD: ICD-9-CM code 296.2–296.36<br><br>Manic disorder: ICD-9-CM code 296.0–296.16<br><br>Personality disorder: ICD-9-CM code 301–301.9<br><br>OCD: ICD-9-CM code 300.3<br><br>Phobic disorder: ICD-9-CM code 300.20–300.29<br><br>Anxiety: ICD-9-CM code 300.0–300.09	10.03	9
Chia 2005[8]	Prospective cohort	49	United States	NR	74.6	Depression: Center for Epidemiologic Studies Depression Scale (CES-D)<br><br>Suicide ideation: evaluated for suicidal ideation and interviewed using the mood dis-<br>orders portion of the Structured Clinical Interview for DSM-IV Axis I Disorders.	0.25	8
Cohen 2007[9]	Prospective cohort	99	Canada	22	33	Depression: Center for Epidemiologic Studies Depression Scale (CES-D)	0.083	5
Droitcourt 2020[29]	Case series and nested case-control	328018	France	NR	56	Suicide: ICD-10 codes X60.x–X69.x, X70.x–X79.x, X80.x–X84.x, Y87.0, Y10.x–Y34.x and R99.<br><br>Suicide attempt: ICD-10 codes X60.x–X69.x, X70.x–X79.x and X80.x–X84.x.	0.33	9
Ferahbas 2004[11]	Prospective cohort	45	Turkey	20.26	56.5	Suicidal thoughts reported as part of Montgomery Asberg Depression Rating Scale (MADRS)	0.31	5

© 2023 Tan NKW et al. *JAMA Dermatology*.

Jick 2000 (Canada)[13]	Retrospective cohort	7195	Canada	NR	53	Neuropsychotic disorders: ICD-9 codes 296-301<br><br>Suicide or attempted suicide: ICD-9, E code from the accident field	NR	8
Jick 2000 (UK)[13]	Retrospective cohort	340	United Kingdom	NR	58	Neuropsychotic disorders: ICD-8 codes 306-311<br><br>Suicide: Oxford Medical Information System code 3009D<br><br>Attempted suicide: Oxford Medical Information System codes 3009C, 3009BN, 3009BT, 3009BP, 9779DL, 9779NA, and 9779L	NR	8
Kaymak 2006[14]	Prospective cohort	100	Turkey	24.4	42	Depression: Turkish version of Hamilton Depression Rating Scale	0.5	5
Kridin 2022[15]	Retrospective cohort	Depression: 73658<br><br>Anxiety: 65735<br>Psychotic disorder: 75,603<br><br>Bipolar disorder: 74,887	Multinational (countries not specified)	21.7	48.3	NR	NR	7
Metekoglu 2019[16]	Prospective cohort	55	Turkey	22	15.3	Depression and anxiety: Hospital Anxiety Depression scale (HAD)	NR	5
Nevoralova 2013[17]	Prospective cohort	100	Czech Republic	18.1	71	Suicide ideation: Beck Depression Inventory, Version II (BDI-II)	0.75	5
Nikam 2020[18]	Prospective cohort	300	India	27.7	55.3	Depression: Hamilton Anxiety rating scale (HAM-A)<br><br>Anxiety: Montgomery Asberg Depression Rating Scale (MADRS)	0.25	5
Paljarvi 2022[19]	Retrospective cohort	All neuropsychiatric: 46974<br><br>Anxiety: 51667<br><br>Psychotic disorder: 56721<br><br>Sleep disorder: 54947	Multinational (91% from United States)	18	53	Mood disorders: ICD-10-CM F30-F39<br><br>Anxiety disorders: ICD-10-CM F40-F48<br><br>Behavioural disorders: ICD-10-CM F10-F19<br><br>Psychotic disorders: ICD-10-CM F20-F29<br><br>Sleep disorders: ICD-10-CM F51, G47<br><br>Personality disorders: ICD-10-CM F60-F69	1	7
Rademaker 2010[20]	Retrospective cohort	1743	New Zealand	23.2	47.4	NR	0.25	5
Rehn 2009[21]	Prospective cohort	109	Finland	20	100	Depression and suicidal ideation: Beck Depression Inventory (BDI)	0.25	5
Singer 2019[22]	Retrospective cohort	444995	United States	22.1	50.1	NR	NR	4

© 2023 Tan NKW et al. *JAMA Dermatology*.

Soundararajan 2019[23]	Retrospective cohort	1087	United States	27	NR	Depression:<br>ICD-9 and 10 codes 296.2, 296.3, 311 and F32, F33	NR	7
Sundström 2010[24]	Retrospective cohort	5756	Sweden	24.09	63	Suicide:<br>ICD-8 codes E950-E958 and E980-E988<br>ICD-9 codes E950-E958 and E980-E988<br>ICD-10 codes X-60-X64 and Y10-Y34	NR	8
Tahir 2011[25]	Prospective cohort	248	Pakistan	21.28	44.8	NR	0.31	5
Ugonabo 2021[26]	Retrospective cohort	26041	United States	23.8	51	Suicide:<br>ICD-9 codes E950-958, V62.84<br>ICD-10 codes T14.91xa, X71-X83, R45.851	1	7
Vona-Giralt 2022[27]	Retrospective cohort	4738	Spain	23.2	0	Anxiety disorders: F40-F41<br>Bipolar and mood disorders: F30-F31, F34, F38, F39<br>Depressive disorders: F32-F33<br>Eating disorders: F50<br>Hallucination/emotional alteration: R44, R45.0-R45.7<br>Insomnia: F51.0, G47.0, G47.9<br>Obsessive-compulsive disorder: F42<br>Personality disorders: F60-F61<br>Psychotic disorders: F20-F23, F25, F28-F29<br>Self-injurious behaviour/suicidal ideation: X60-X84, R45.8<br>Somatomorph/neurotic disorders: F45, F48	0.53	7

© 2023 Tan NKW et al. *JAMA Dermatology*.

eTable 5B. Summary of Included Studies for Relative Risk of Psychiatric Disorders

Study	Study design	Analytical sample size	Country	Mean age	% Male	Outcome definition	Covariates	Mean follow-up duration/ years	NOS Score
Azoulay 2008[28]	Retrospective case cross-over	126	Canada	NR	NR	Depression: ICD-9 codes 296.2, 298.0, 300.4, 309.0, 309.1, and 311	NR	0.42	8
Chen 2022[7]	Retrospective cohort	9981	Taiwan	38.0	34.58	Suicidality: ICD-9-CM code E950–E959<br><br>ADHD: ICD-9-CM code 314.0–314.01<br><br>Schizophrenia: ICD-9-CM code 295–295.95<br><br>BD: ICD-9-CM code 296.4–296.7<br><br>MDD: ICD-9-CM code 296.2–296.36<br><br>Manic disorder: ICD-9-CM code 296.0–296.16<br><br>Personality disorder: ICD-9-CM code 301–301.9<br><br>OCD: ICD-9-CM code 300.3<br><br>Phobic disorder: ICD-9-CM code 300.20–300.29<br><br>Anxiety: ICD-9-CM code 300.0–300.09	Age, gender, Charlson Comorbidity Index	10.03	9
Droitcourt 2019[10]	Retrospective cohort and nested case-time-control	443814	France	20	55	Suicide attempt: ICD-10 codes X60.x to X69.x, X70.x to X79.x and X80.x to X84.x.	Age, gender, month and calender year	5	7
Gupta 2020[12]	Retrospective cohort	218594	United States	22.66	NR	Insomnia: MedDRA code: 10022437, 10022035, 10027590, 10068932	NR	NR	4
Jick 2000 (Canada)[3]	Retrospective cohort	7195	Canada	NR	53	Neuropsychotic disorders: ICD-9 codes 296-301	History of psychiatric disorder, age (same 10-year age band)	NR	8

© 2023 Tan NKW et al. *JAMA Dermatology*.

						Suicide or attempted suicide: ICD-9, E code from the accident field			
Jick 2000 (UK)[3]	Retrospective cohort	340	United Kingdom	NR	58	Neuropsychotic disorders: ICD-8 codes 306-311<br><br>Suicide: Oxford Medical Information System code 3009D<br><br>Attempted suicide: Oxford Medical Information System codes 3009C, 3009BN, 3009BT, 3009BP, 9779DL, 9779NA, and 9779L	History of psychiatric disorder, age (same 10-year age band)	NR	8
Kridin 2022[15]	Retrospective cohort	Depression: 73658<br><br>Anxiety: 65735<br><br>Psychotic disorder: 75,603<br><br>Bipolar disorder: 74,887	Multinational (countries not specified)	21.7	48.3	NR	NR	NR	7
Paljarvi 2022[19]	Retrospective cohort	All neuropsychiatric: 46974<br><br>Anxiety: 51667<br><br>Psychotic disorder: 56721<br><br>Sleep disorder: 54947	Multinational (91% from United States)	18	53	Mood disorders: ICD-10-CM F30-F39<br><br>Anxiety disorders: ICD-10-CM F40-F48<br><br>Behavioural disorders: ICD-10-CM F10-F19<br><br>Psychotic disorders: ICD-10-CM F20-F29<br><br>Sleep disorders: ICD-10-CM F51, G47<br><br>Personality disorders: ICD-10-CM F60-F69	Age at index prescription or event; sex; ethnicity/race; mental and behavioural disorders because of psychoactive substance use; schizophrenia, schizotypal, delusional and other non-mood psychotic disorders; mood (affective) disorders; anxiety, dissociative, stress-related, somatoform and other nonpsychotic mental disorders; sleep disorders not because of a substance or known physiological condition; disorders of adult personality and behaviour; behavioural and emotional disorders with onset usually occurring in childhood and adolescence; sleep disorders; vasomotor and allergic rhinitis; asthma; dermatitis and eczema; acne	1	7

© 2023 Tan NKW et al. *JAMA Dermatology*.

							vulgaris; acne conglobata; other acne; unspecified acne; suicidal ideations; suicide attempt; intentional self-harm; and dispensed prescriptions for sedatives and hypnotics; antidepressants; antipsychotics; and glucocorticoids.		
Sundström 2010[24]	Retrospective cohort	5756	Sweden	24.09	63	Suicide:<br>ICD-8 codes E950-E958 and E980-E988<br>ICD-9 codes E950-E958 and E980-E988<br>ICD-10 codes X-60-X64 and Y10-Y34	Age, sex, calendar year	NR	8
Vona-Giralt 2022[27]	Retrospective cohort	4738	Spain	23.2	0	Anxiety disorders: F40-F41<br>Bipolar and mood disorders: F30-F31, F34, F38, F39<br>Depressive disorders: F32-F33<br>Eating disorders: F50<br>Hallucination/emotional alteration: R44, R45.0-R45.7<br>Insomnia: F51.0, G47.0, G47.9<br>Obsessive-compulsive disorder: F42<br>Personality disorders: F60-F61<br>Psychotic disorders: F20-F23, F25, F28-F29<br>Self-injurious behaviour/suicidal ideation: X60-X84, R45.8<br>Somatomorph/neurotic disorders: F45, F48	Age	0.53	7

© 2023 Tan NKW et al. *JAMA Dermatology*.

eTable 5C. Summary of Included Studies for Risk Factors of Psychiatric Disorders

Study	Study design	Analytical sample size	Country	Mean age	% Male	Outcome definition	Covariates	Mean follow-up duration/ years	NOS Score
Chen 2022[7]	Retrospective cohort	9981	Taiwan	38.0	34.58	Suicidality: ICD-9-CM code E950–E959<br><br>ADHD: ICD-9-CM code 314.0–314.01<br><br>Schizophrenia: ICD-9-CM code 295–295.95<br><br>BD: ICD-9-CM code 296.4–296.7<br><br>MDD: ICD-9-CM code 296.2–296.36<br><br>Manic disorder: ICD-9-CM code 296.0–296.16<br><br>Personality disorder: ICD-9-CM code 301–301.9<br><br>OCD: ICD-9-CM code 300.3<br><br>Phobic disorder: ICD-9-CM code 300.20–300.29<br><br>Anxiety: ICD-9-CM code 300.0–300.09	Age, gender, Charlson Comorbidity Index	10.03	9
Droitcourt 2020[29]	Case series and nested case-control	328018	France	NR	56	Suicide: ICD-10 codes X60.x–X69.x, X70.x–X79.x, X80.x–X84.x, Y87.0, Y10.x–Y34.x and R99.<br><br>Suicide attempt: ICD-10 codes X60.x–X69.x, X70.x–X79.x and X80.x–X84.x.	Age, area of residence	0.33	9
Vona-Giralt 2022[27]	Retrospective cohort	4738	Spain	23.2	0	Anxiety disorders: F40-F41<br>Bipolar and mood disorders: F30-F31, F34, F38, F39<br>Depressive disorders: F32-F33<br>Eating disorders: F50<br>Hallucination/emotional alteration: R44, R45.0-R45.7<br>Insomnia: F51.0, G47.0, G47.9	Age	0.53	7

© 2023 Tan NKW et al. *JAMA Dermatology*.

						Obsessive-compulsive disorder: F42<br>Personality disorders: F60-F61<br>Psychotic disorders: F20-F23, F25, F28-F29<br>Self-injurious behaviour/suicidal ideation: X60-X84, R45.8<br>Somatomorph/neurotic disorders: F45, F48			

© 2023 Tan NKW et al. *JAMA Dermatology*.

eTable 6. Meta-Regression of Prespecified Study Level Characteristics for 1-Year Absolute Risk of Depression

	**Beta‡**	**SE**	**Z**	**P**	**95% CI Lower**	**95% CI Upper**	**R2 (% heterogeneity accounted for)**	**Permutation testing p-value**
Average age	-0.152865	0.06422519	-2.3801405	**0.01730604**	-0.278744	-0.0269859	41.9363167	0.026
% Male	0.00142834	0.01060906	0.13463434	0.89290099	-0.019365	0.02222173	0	-
Average follow-up duration	0.18978445	2.09581163	0.09055416	0.92784686	-3.9179309	4.29749978	0	-
NOS score	-0.156669	0.21750034	-0.7203163	0.47133028	-0.5829619	0.2696238	0	-
Study design (prospective vs retrospective)	-0.4141633	0.48306439	-0.8573666	0.39124233	-1.3609521	0.53262555	0	-

© 2023 Tan NKW et al. *JAMA Dermatology*.

eTable 7. Meta-Regression of Prespecified Study-Level Characteristics for 1-Year Absolute Risk of Completed Suicide

	**Beta‡**	**SE**	**Z**	**P**	**95% CI Lower**	**95% CI Upper**	**R2 (% heterogeneity accounted for)**	**Permutation testing p-value**
Average age	-0.57367268	0.521138315	-1.100806951	0.270980686	-1.59508501	0.447739649	5.196308582	-
% Male	0.163290034	0.080027159	2.040432729	0.041307245	0.006439685	0.320140383	38.59601551	0.026
Average follow-up duration	-3.087098801	3.128324924	-0.986821662	0.323730073	-9.218502984	3.044305383	0	-
NOS score	-0.116734985	0.446495591	-0.261447118	0.793747726	-0.991850264	0.758380293	0	-
Study design (prospective vs retrospective)	-3.56786356	1.226678154	-2.908557187	0.003631008	-5.972108561	-1.163618558	59.273716	0.026

© 2023 Tan NKW et al. *JAMA Dermatology*.

eFigure 1. Meta-Analysis for 1-Year Absolute Risk of All Psychiatric Disorders, Anxiety, Psychotic Disorders, and Sleep Disorders

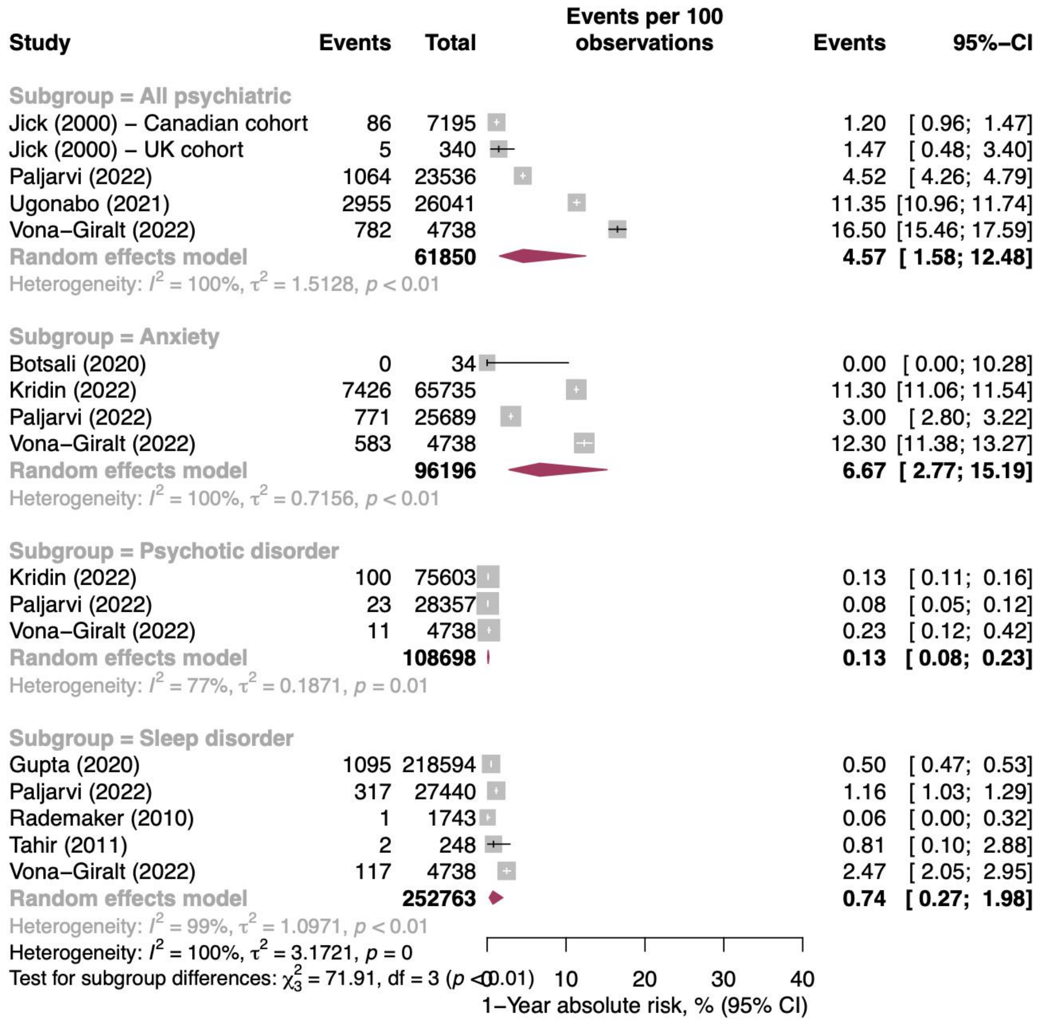

© 2023 Tan NKW et al. *JAMA Dermatology*.

eFigure 2. Meta-Regression for 1-Year Absolute Risk of Depression (Average Age)

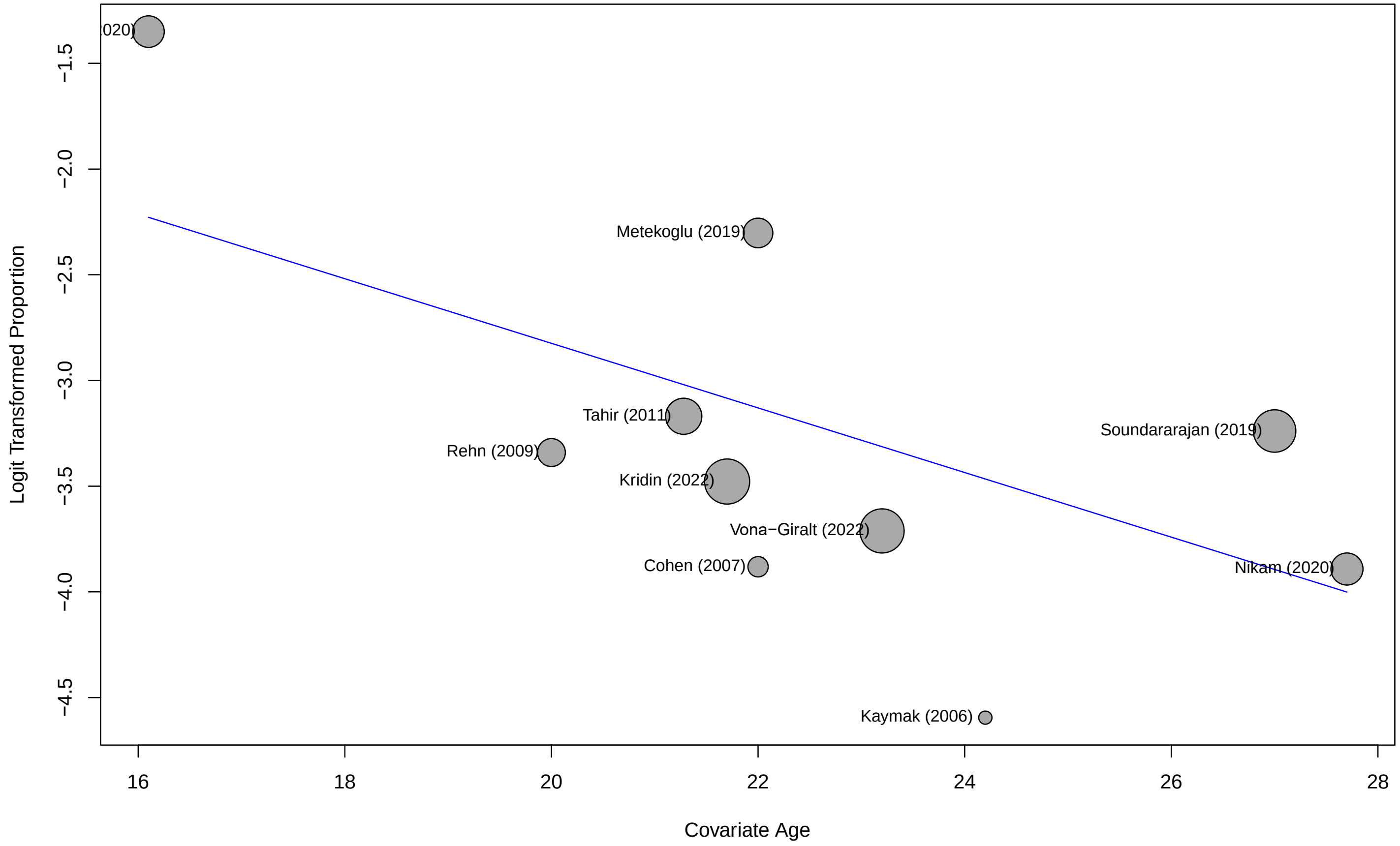

© 2023 Tan NKW et al. *JAMA Dermatology*.

eFigure 3. Meta-Regression for 1-Year Absolute Risk of Completed Suicide (Sex)

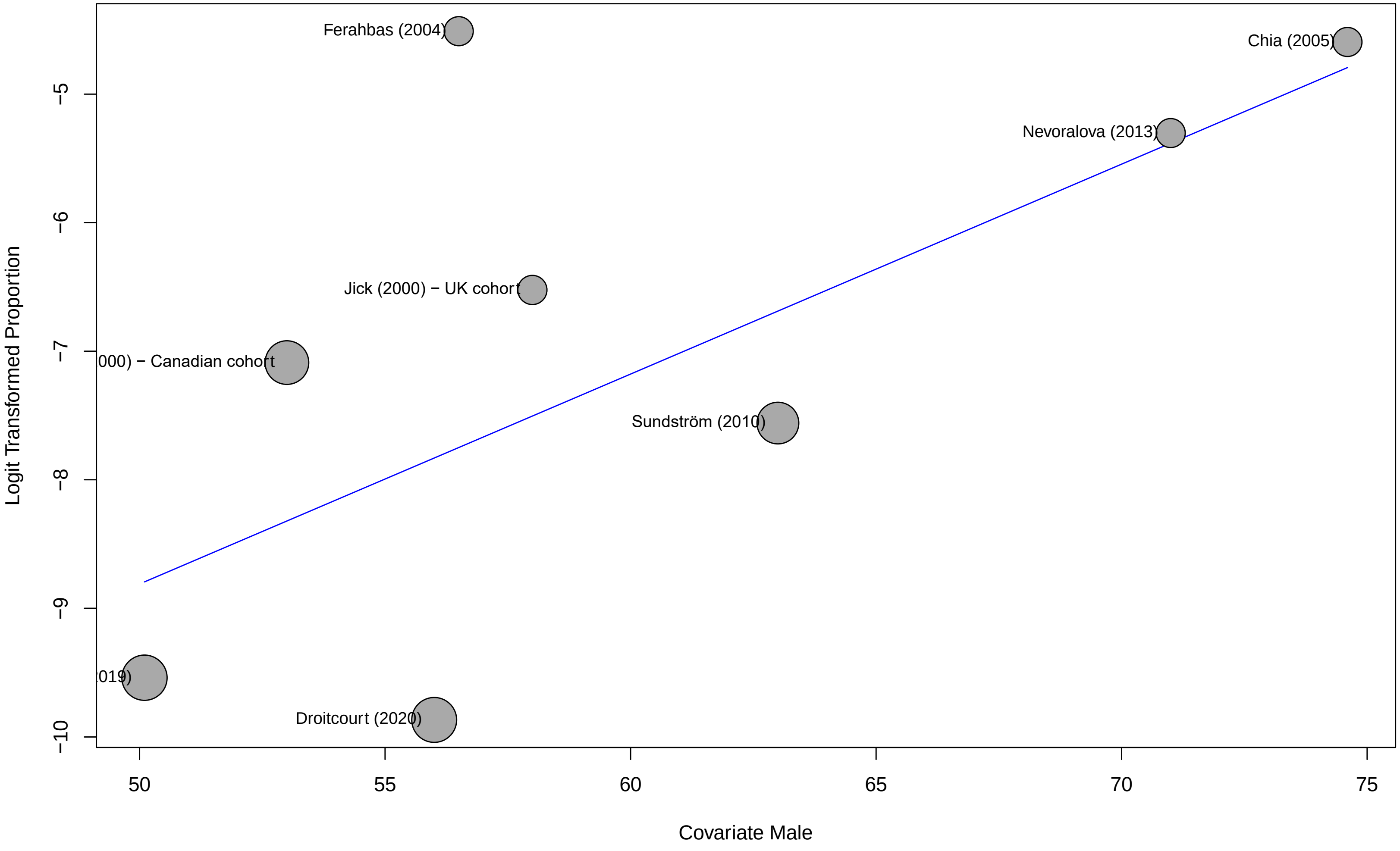

© 2023 Tan NKW et al. *JAMA Dermatology*.

eFigure 4. Meta-Regression for 1-Year Absolute Risk of Completed Suicide (Study Design)

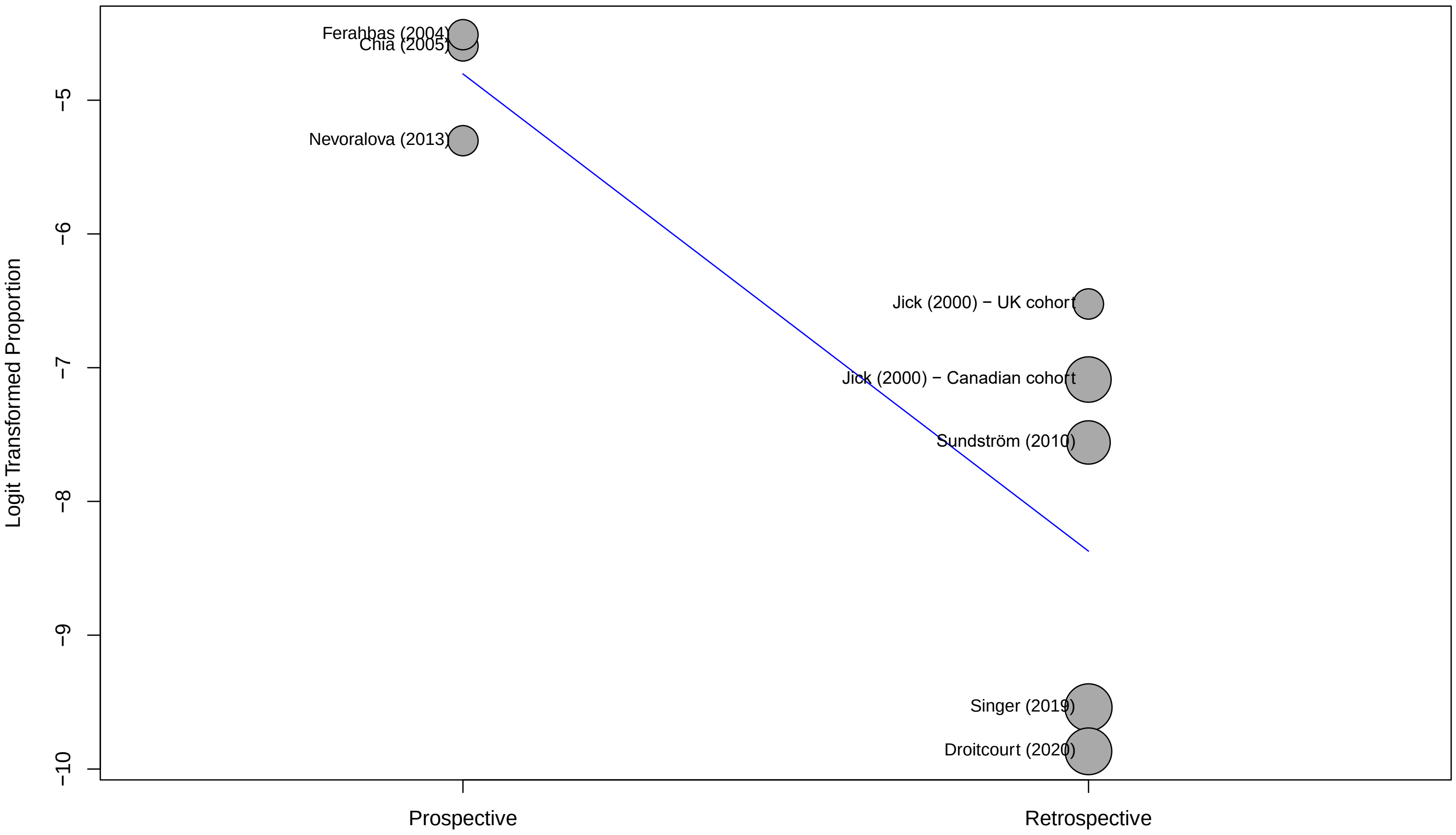

© 2023 Tan NKW et al. *JAMA Dermatology*.

eFigure 5. Contour-Enhanced Funnel Plots, With Missing Studies Imputed Via the Trim-and-Fill Method

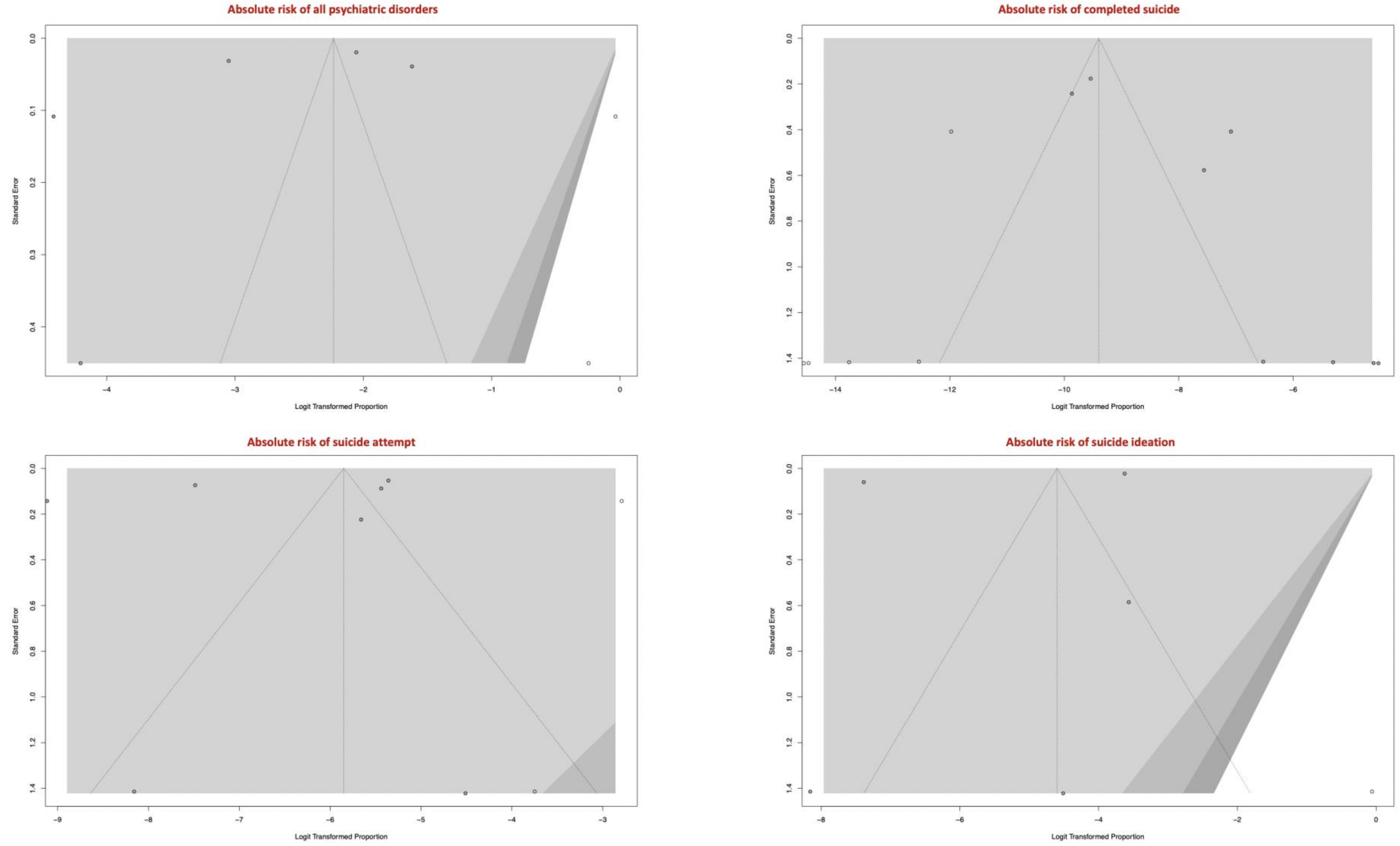

© 2023 Tan NKW et al. *JAMA Dermatology*.

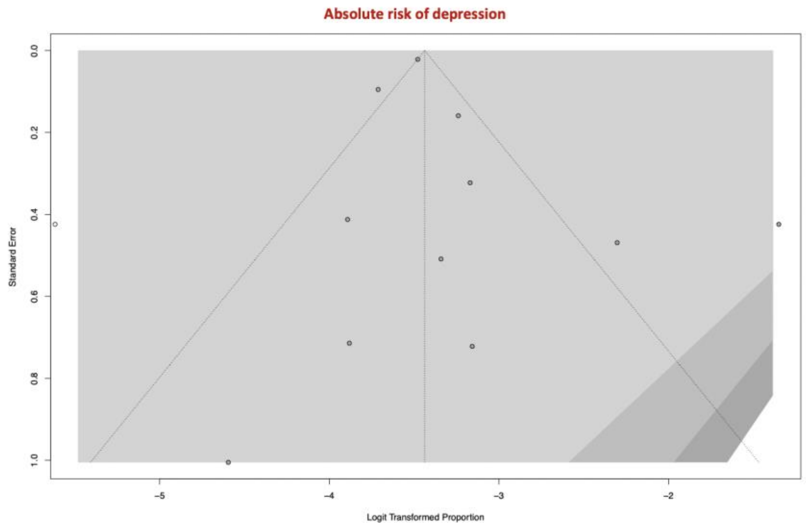

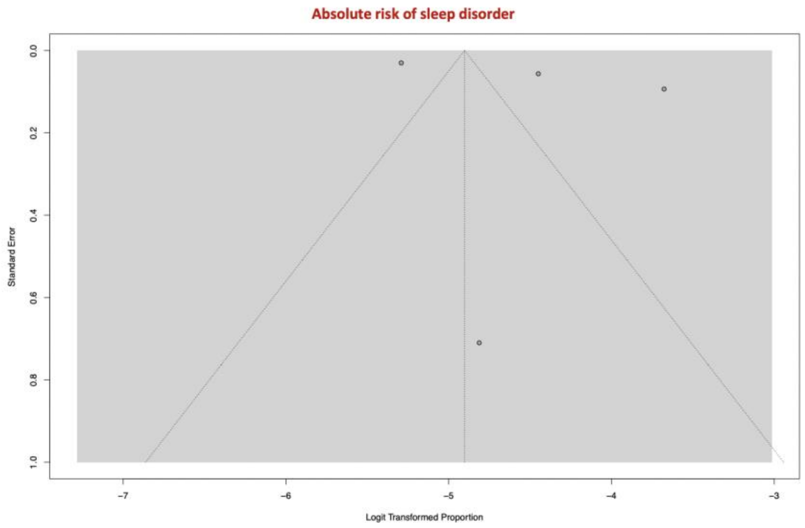

Egger's test was performed for absolute risk of depression, and results indicated no publication bias (intercept= -3.50; t=0.91; p=0.38).

© 2023 Tan NKW et al. *JAMA Dermatology*.

eFigure 6. Meta-Analysis for Relative Risk of All Psychiatric Disorders, Depression, Anxiety, Psychotic Disorders, and Sleep Disorders

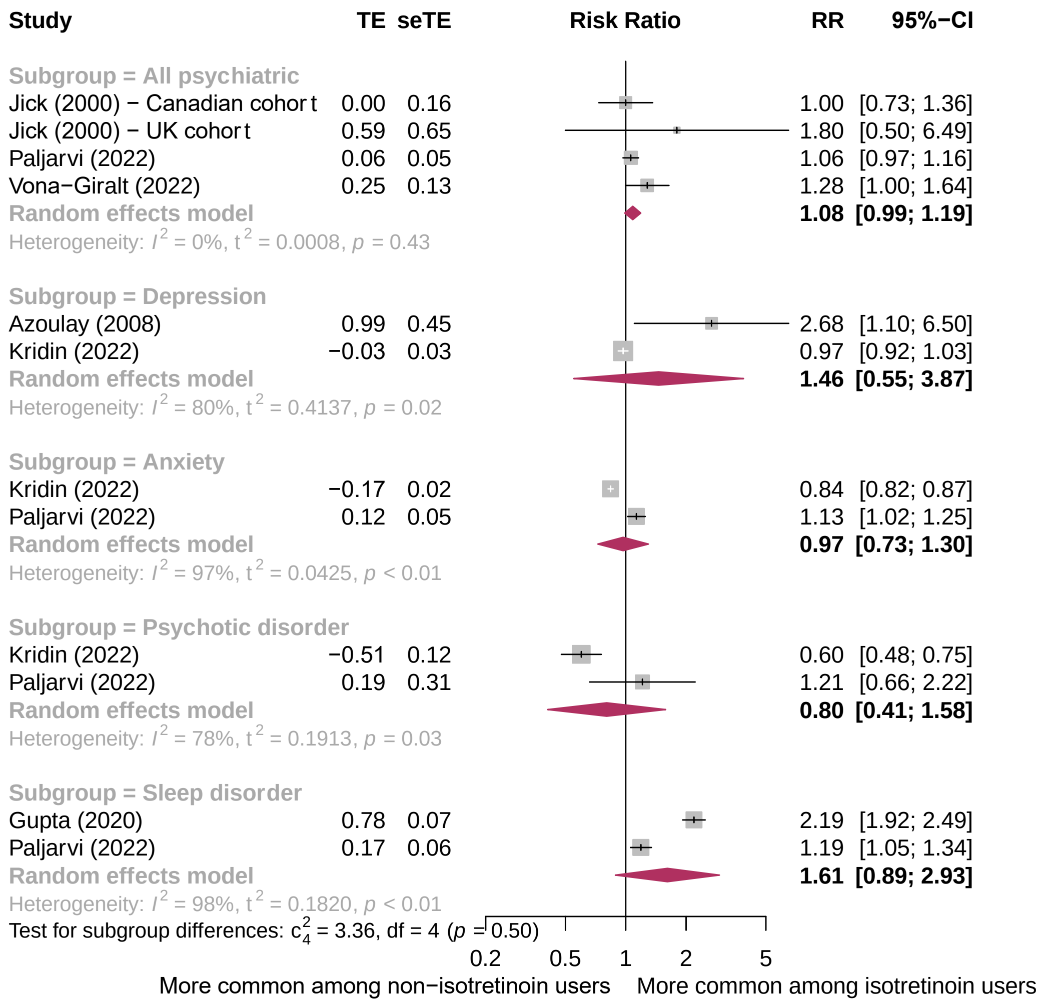

© 2023 Tan NKW et al. *JAMA Dermatology*.

eReferences

1. Moher D, Liberati A, Tetzlaff J, Altman DG, The PG. Preferred Reporting Items for Systematic Reviews and Meta-Analyses: The PRISMA Statement. *PLOS Medicine.* 2009;6(7):e1000097.
2. Stroup DF, Berlin JA, Morton SC, et al. Meta-analysis of Observational Studies in EpidemiologyA Proposal for Reporting. *JAMA.* 2000;283(15):2008-2012.
3. GA Wells BS, D O'Connell, J Peterson, V Welch, M Losos, P Tugwell,. The Newcastle-Ottawa Scale (NOS) for assessing the quality of nonrandomised studies in meta-analyses. http://www.ohri.ca/programs/clinical_epidemiology/oxford.asp. Published 2012. Accessed April 24, 2019.
4. Cochrane Collaboration. Section 13.5.2.3. Tools for assessing methodological quality or risk of bias in non-randomized studies. In: Higgins JPT, Green S, eds. *Cochrane Handbook for Systematic Reviews of Interventions, Version 5.1.0.* London2011.
5. Botsali A, Kocyigit PP, Uran P. The effects of isotretinoin on affective and cognitive functions are disparate in adolescent acne vulgaris patients. *J Dermatolog Treat.* 2020;31(7):734-738.
6. Brzezinski P, Borowska K, Chiriac A, Smigielski J. Adverse effects of isotretinoin: A large, retrospective review. *Dermatol Ther.* 2017;30(4).
7. Chen YH, Wang WM, Chung CH, Tsao CH, Chien WC, Hung CT. Risk of psychiatric disorders in patients taking isotretinoin: A nationwide, population-based, cohort study in Taiwan. *J Affect Disord.* 2022;296:277-282.
8. Chia CY, Lane W, Chibnall J, Allen A, Siegfried E. Isotretinoin therapy and mood changes in adolescents with moderate to severe acne: a cohort study. *Arch Dermatol.* 2005;141(5):557-560.
9. Cohen J, Adams S, Patten S. No association found between patients receiving isotretinoin for acne and the development of depression in a Canadian prospective cohort. *Can J Clin Pharmacol.* 2007;14(2):e227-233.
10. Droitcourt C, Nowak E, Rault C, et al. Risk of suicide attempt associated with isotretinoin: a nationwide cohort and nested case-time-control study. *Int J Epidemiol.* 2019;48(5):1623-1635.
11. Ferahbas A, Turan MT, Esel E, Utas S, Kutlugun C, Kilic CG. A pilot study evaluating anxiety and depressive scores in acne patients treated with isotretinoin. *J Dermatolog Treat.* 2004;15(3):153-157.
12. Gupta MA, Vujcic B, Gupta AK. 13711 Isotretinoin use in acne is associated with a higher odds of the adverse effect of insomnia: Results from the US FDA Adverse Events Reporting System. *Journal of the American Academy of Dermatology.* 2020;83(6):AB121.
13. Jick SS, Kremers HM, Vasilakis-Scaramozza C. Isotretinoin use and risk of depression, psychotic symptoms, suicide, and attempted suicide. *Arch Dermatol.* 2000;136(10):1231-1236.
14. Kaymak Y, Kalay M, Ilter N, Taner E. Incidence of depression related to isotretinoin treatment in 100 acne vulgaris patients. *Psychol Rep.* 2006;99(3):897-906.

© 2023 Tan NKW et al. *JAMA Dermatology*.

15. Kridin K, Ludwig RJ. Isotretinoin and the risk of psychiatric disturbances: A global study shedding new light on a debatable story. *Journal of the American Academy of Dermatology.* 2023;88(2):388-394.
16. Metekoglu S, Oral E, Ucar C, Akalin M. Does isotretinoin cause depression and anxiety in acne patients? *Dermatol Ther.* 2019;32(2):e12795.
17. Nevoralová Z, Dvořáková D. Mood changes, depression and suicide risk during isotretinoin treatment: a prospective study. *Int J Dermatol.* 2013;52(2):163-168.
18. Nikam B, Jamale V, Ravikumar ST. Effect of Oral Isotretinoin on Anxiety and Depression in Patients with Acne. *Journal of Pharmacology and Pharmacotherapeutics.* 2020;11(3):113-118.
19. Paljarvi T, McPherson T, Luciano S, Herttua K, Fazel S. Isotretinoin and adverse neuropsychiatric outcomes: retrospective cohort study using routine data. *Br J Dermatol.* 2022;187(1):64-72.
20. Rademaker M. Adverse effects of isotretinoin: A retrospective review of 1743 patients started on isotretinoin. *Australasian Journal of Dermatology.* 2010;51(4):248-253.
21. Rehn LM, Meririnne E, Höök-Nikanne J, Isometsä E, Henriksson M. Depressive symptoms and suicidal ideation during isotretinoin treatment: a 12-week follow-up study of male Finnish military conscripts. *J Eur Acad Dermatol Venereol.* 2009;23(11):1294-1297.
22. Singer S, Tkachenko E, Sharma P, Barbieri JS, Mostaghimi A. Psychiatric Adverse Events in Patients Taking Isotretinoin as Reported in a Food and Drug Administration Database From 1997 to 2017. *JAMA Dermatol.* 2019;155(10):1162-1166.
23. Vinaya Soundararajan EG, Rachel Lefferdink, Bethanee Schlosser, Dennis P. West, Beatrice Nardone. Frequency of depression in dermatologist-managed patients who have acne, isotretinoin-exposure vs no isotretinoin exposure: Pharmacovigilance analysis of a large Midwestern US population from the RADAR (Research on Adverse Drug events And Reports) program. *Journal of the American Academy of Dermatology.* 2019;81(4):AB67.
24. Sundström A, Alfredsson L, Sjölin-Forsberg G, Gerdén B, Bergman U, Jokinen J. Association of suicide attempts with acne and treatment with isotretinoin: retrospective Swedish cohort study. *Bmj.* 2010;341:c5812.
25. Tahir CM. Efficacy and adverse effects of systemic isotretinoin therapy. *Journal of Pakistan Association of Dermatologists.* 2011;21:38-42.
26. Ugonabo N, Love E, Wong PW, et al. Psychiatric disorders and suicidal behavior in patients with acne prescribed oral antibiotics versus isotretinoin: Analysis of a large commercial insurance claims database. *J Am Acad Dermatol.* 2021;85(4):878-884.
27. Vona-Giralt G, Vilaplana-Carnerero C, Ouchi D, Gomez-Lumbreras A, Morros R, Giner-Soriano M. Risk of psychiatric events in women treated with isotretinoin: a self-controlled study with SIDIAP database. *Expert Opin Drug Saf.* 2022:1-7.
28. Azoulay L, Blais L, Koren G, LeLorier J, Bérard A. Isotretinoin and the risk of depression in patients with acne vulgaris: a case-crossover study. *J Clin Psychiatry.* 2008;69(4):526-532.

© 2023 Tan NKW et al. *JAMA Dermatology*.

29. Droitcourt C, Poizeau F, Kerbrat S, et al. Isotretinoin and risk factors for suicide attempt: a population-based comprehensive case series and nested case–control study using 2010–2014 French Health Insurance Data. *Journal of the European Academy of Dermatology and Venereology.* 2020;34(6):1293-1301.
30. Guyatt GH, Oxman AD, Vist GE, et al. GRADE: an emerging consensus on rating quality of evidence and strength of recommendations. *BMJ.* 2008;336(7650):924.

© 2023 Tan NKW et al. *JAMA Dermatology*.
