# Supplementary material for: Risk of Suicide and Psychiatric Disorders Among Isotretinoin Users: A Meta-Analysis
Source: JAMA Dermatol. 2023 Nov 29;160(1):54–62. doi: 10.1001/jamadermatol.2023.4579 (PMC10687715; doi:10.1001/jamadermatol.2023.4579)
Supplement: Supplement 2. — Data Sharing Statement [file jamadermatol-e234579-s002.pdf]

Data Sharing Statement

Tan. Risk of Suicide and Psychiatric Disorders Among Isotretinoin Users. *JAMA Dermatol*. Published November 29, 2023. doi:10.1001/jamadermatol.2023.4579

Data
Data available: No

Additional Information
Explanation for why data not available: As this is a meta-analysis of published data, we do not have access to individual patient data. However, the data for this meta-analysis can be made available on reasonable request to the corresponding author.
